# Characteristics and immune functions of the endogenous CRISPR-Cas systems in myxobacteria

**DOI:** 10.1128/msystems.01210-23

**Published:** 2024-05-15

**Authors:** Wei-feng Hu, Jiang-yu Yang, Jing-jing Wang, Shu-fei Yuan, Xin-jing Yue, Zheng Zhang, Ya-qi Zhang, Jun-yan Meng, Yue-zhong Li

**Affiliations:** 1 State Key Laboratory of Microbial Technology, Institute of Microbial Technology, Shandong University, Qingdao, China; Danmarks Tekniske Universitet, Kgs. Lyngby, Lyngby-Taarbæk, Denmark

**Keywords:** CRISPR-Cas, myxobacteria, *Myxococcus xanthus*, target tracing, immunity

## Abstract

The clustered regularly interspaced short palindromic repeats and their associated proteins (CRISPR-Cas) system widely occurs in prokaryotic organisms to recognize and destruct genetic invaders. Systematic collation and characterization of endogenous CRISPR-Cas systems are conducive to our understanding and potential utilization of this natural genetic machinery. In this study, we screened 39 complete and 692 incomplete genomes of myxobacteria using a combined strategy to dispose of the abridged genome information and revealed at least 19 CRISPR-Cas subtypes, which were distributed with a taxonomic difference and often lost stochastically in intraspecies strains. The *cas* genes in each subtype were evolutionarily clustered but deeply separated, while most of the CRISPRs were divided into four types based on the motif characteristics of repeat sequences. The spacers recorded in myxobacterial CRISPRs were in high G+C content, matching lots of phages, tiny amounts of plasmids, and, surprisingly, massive organismic genomes. We experimentally demonstrated the immune and self-target immune activities of three endogenous systems in *Myxococcus xanthus* DK1622 against artificial genetic invaders and revealed the microhomology-mediated end-joining mechanism for the immunity-induced DNA repair but not homology-directed repair. The panoramic view and immune activities imply potential omnipotent immune functions and applications of the endogenous CRISPR-Cas machinery.

**IMPORTANCE:**

Serving as an adaptive immune system, clustered regularly interspaced short palindromic repeats and their associated proteins (CRISPR-Cas) empower prokaryotes to fend off the intrusion of external genetic materials. Myxobacteria are a collective of swarming Gram-stain-negative predatory bacteria distinguished by intricate multicellular social behavior. An in-depth analysis of their intrinsic CRISPR-Cas systems is beneficial for our understanding of the survival strategies employed by host cells within their environmental niches. Moreover, the experimental findings presented in this study not only suggest the robust immune functions of CRISPR-Cas in myxobacteria but also their potential applications.

## INTRODUCTION

Clustered regularly interspaced short palindromic repeats (CRISPR) and their associated proteins (Cas) constitute the RNA-mediated adaptive immune systems in prokaryotes. A CRISPR-Cas system constitutes a *cas* gene cluster and an often neighboring CRISPR array with a short leader sequence separation in front of the array (1). The CRISPR array stores short sequences of previously captured genetic invaders as spacers, which are separated with intervals of repeat sequences. The mature RNAs from CRISPR (crRNAs) guide the Cas proteins or complex to recognize and destroy the matched foreign nucleotide sequences or mediate abortive infection responses (2–4). The immune function of the CRISPR-Cas system is realized in three stages. The adaptation stage captures new spacer sequences from intruders, achieved by a naive process or a priming process (5), and integrates them into CRISPR at the beginning of the array, providing a chronological record of past infections. The second stage is the maturation of small crRNAs from a long proto-RNA sequence transcribed from the CRISPR array. In the interference stage, crRNA assembles with defined Cas proteins into a ribonucleoprotein complex, which recognizes and degrades targeted invasion sequences under the guidance of crRNA (6). The protospacer in intruders normally has a short (3–6 nt) adjacent sequence element, named protospacer adjacent motif (PAM), which is critical for the generation and/or integration of spacers into CRISPR, as well as the recognition and degradation of target DNA. Although linked to many cellular functions like gene regulation, biofilm formation, multicellular development, and virulence (7), the fundamental role of CRISPR-Cas systems is the immunization of cells from genetic invaders. Systematic collation and comprehensive characterization of endogenous CRISPR-Cas systems can aid to understand the host survival strategies, as well as to develop genetic tools with them, especially for the genetic manipulation in non-model bacteria (8).

The CRISPR-Cas system has evolved into diverse types, presently containing 2 classes, 6 types, and over 30 subtypes, mainly depending on the phylogeny of signature proteins and the architecture of *cas* genes (9). Notably, many new (sub)types were recently revealed from genome mining with unknown characteristics (10). Up to date, some CRISPR-Cas systems have been systematically explored in some prokaryotic species, such as *Escherichia coli* (11), *Sulfolobus islandicus* (12), *Lactobacillus crispatus* (13), and *Pseudomonas aeruginosa* (14). However, their distribution and characteristics are still unclear in many prokaryotic taxa, mainly due to the lack of complete genome information—many genomes have been incompletely sequenced and released as scaffolds and contigs, and lots of environmental genomes are the assemblies from uncultured metagenomes metagenome assembled genomes (MAGs)—this may lead to incomplete or inaccurate information. It is still a challenge to efficiently summarize and characterize the endogenous CRISPR-Cas (sub)types from incomplete genomes.

Myxobacteria are a group of swarming Gram-stain-negative predatory bacteria and are characterized by their complex multicellular social behavior, such as the cell movement in swarms, predation in groups, and development of multicellular fruiting body structures (15, 16). Myxobacteria are also an intriguing new drug resource (17) and potential biocontrol agents (18, 19). The taxonomy of myxobacteria has been recently upgraded from an order (*Myxococcales*) within the δ division of Proteobacteria to the *Myxococcota* phylum based on 120 conserved single-copy marker genes as well as the rRNA gene (20). The CRISPR-Cas systems in myxobacteria have been previously investigated in the model species *Myxococcus xanthus*, including influences of the genetic loci on the development of fruiting bodies (21, 22) and the production of exopolysaccharides (23), as well as a multifactorial regulation of their transcription (24). However, we know little about the characteristics of endogenous CRISPR-Cas systems, as well as their immune functions in myxobacteria. In addition, although the exotic CRISPR-Cas9 system has been employed in studies of *M. xanthus* (25–27), no application of myxobacterial endogenous CRISPR-Cas systems has been reported.

In this study, we establish a combined pipeline (Fig. S1) to comprehensively analyze the endogenous CRISPR-Cas in myxobacteria, their characteristics, and their potential immunity from a few complete genomes and lots of incomplete genomes. We demonstrated the expression and immune activities of the three CRISPR-Cas systems in *M. xanthus* DK1622 and uncovered the microhomology-mediated end joining (MMEJ) DNA repair mechanism induced by the self-immunity of the endogenous CRISPR-Cas systems, which led to a varied DNA deletion flanking the targeted site. Our work provides a panoramic view of endogenous CRISPR-Cas systems in myxobacteria, and the approach for taxonomically analyzing endogenous CRISPR-Cas systems with incomplete genomes can overcome the limitation of poor sequencing quality. The experimental work also implies that the effectiveness of the endogenous CRISPR-Cas systems is potential for the development of gene manipulation techniques in *M. xanthus*.

## RESULTS

### Occurrence and distribution of CRISPR-Cas systems in myxobacteria

Until 5 January 2022, a total of 731 myxobacterial genomes were available in GenBank; 39 were completely sequenced (containing a repeated genome of *M. xanthus* DZ2), and 692 genomes were incomplete or assembled from metagenomes (Data Set S1). The sequenced myxobacterial genomes were taxonomically uneven (Table S1). Many genera or families, and even the *Nannocystineae* suborder, contained a single sequenced genome, whereas *Myxococcus* had 12 sequenced genomes. Of the 127 myxobacterial genomes with well-defined species classification, the number of *Corallococcus* and *Myxococcus* genomes reached 57 (30 and 27, respectively), accounting for approximately 44.9%.

To gain a comprehensive understanding of the endogenous CRISPR-Cas systems in myxobacteria, we developed a pipeline combining analyses of the Cas proteins and the CRISPRs with the few complete and numerous incomplete genomes (Fig. S1). We first scanned the 38 myxobacterial complete genomes and revealed 53 *cas* gene clusters, belonging to the III-A, III-B, and III-D subtypes and the I-B, MxI-B, I-C, I-E, and I-G subtypes of the class 1 system (Fig. S2; for more details, refer to Data set 2), and this was the same as the count recorded for 47 myxobacterial genomes in CRISPRCasdb (28). Consistent with many other prokaryotes (29), myxobacterial genomes normally had two or more subtypes simultaneously, but some genomes contained single subtypes, like I-C, I-G, I-E, or III-B only, or even lacked the *cas* genes. For example, of the three completely sequenced *Archangium* genomes, while *Archangium violaceum* SDU8 had the single III-B, no *cas* gene cluster was observed in *A. violaceum* SDU34 and *Archangium gephyra* DSM 2261 genomes. For the five *Sorangium cellulosum* genomes, So ce26 had no *cas* gene cluster, while the other four strains possessed one to three *cas* gene clusters.

**Fig 1:**
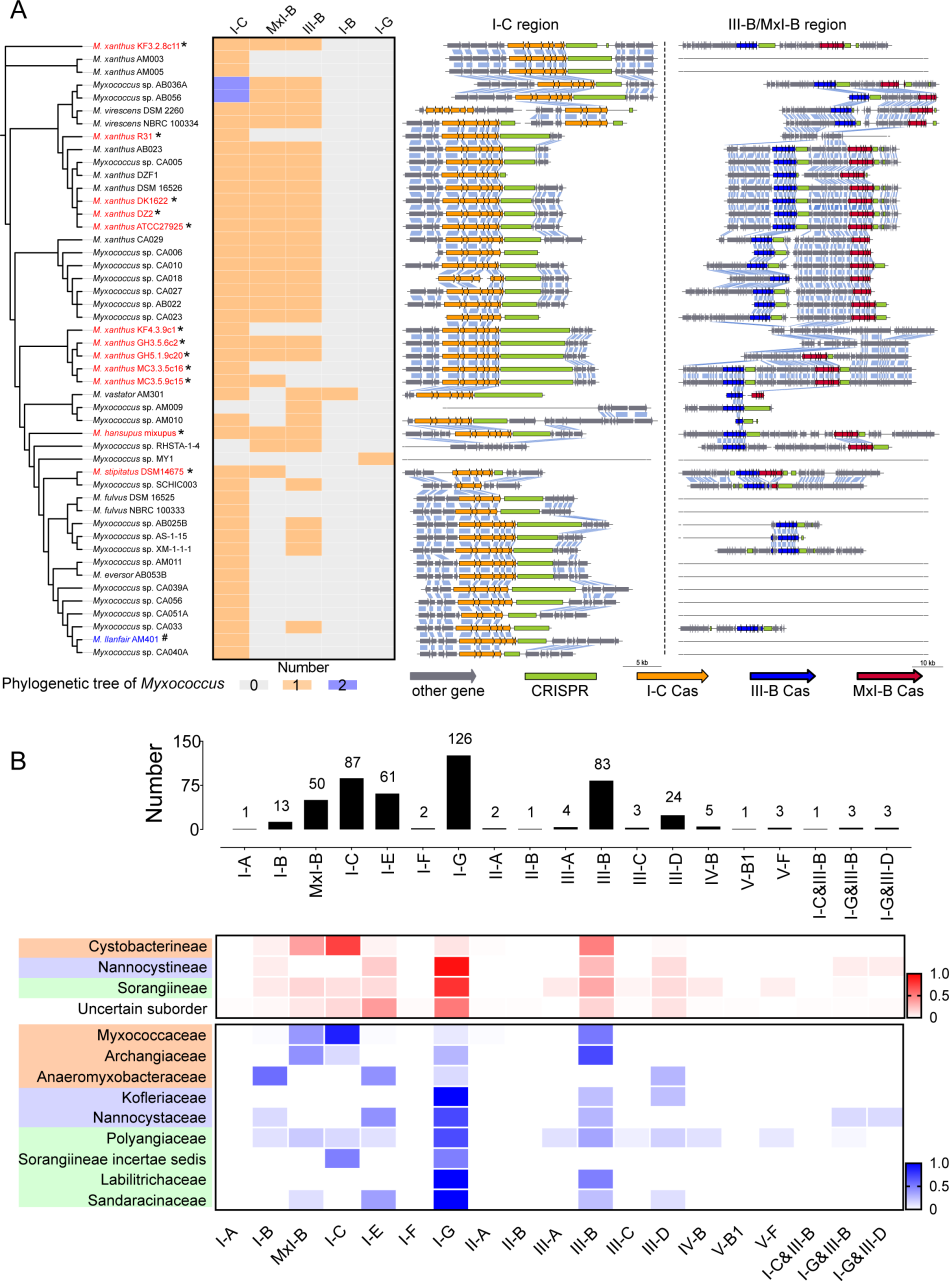
CRISPR-Cas systems and their losses in myxobacteria. (**A**) Distribution and genetic contexts of the CRISPR-Cas systems in *Myxococcus* strains. The phylogenetic tree of *Myxococcus* strains is constructed based on the whole genome, representing the strain clustering, while ignoring the evolutionary distance. The species name of *Myxococcus llanfairpwllgwyngyllgogerychwyrndrobwllllantysiliogogogochensis* AM401 is abbreviated as *M. llanfair* AM401 and is labeled in blue with “#.” Genomes in red with “*” are completely sequenced. The homologous genes before and after the I-C, MxI-B, and III-B clusters in *Myxococcus* were obtained by the context comparison between *Myxococcus* strains. Different scaffolds of a genome are drawn separately. The straight line without any genes means that no similar genetic context is observed. (**B**) Occurrence and distribution of CRISPR-Cas systems in myxobacteria. The upper histogram picture shows the number of the revealed CRISPR-Cas subtypes, and the lower heat map shows the distribution of CRISPR-Cas subtypes in different myxobacterial taxa. The heat map in red is the distribution at the suborder level, and the heat map in blue shows the distribution under the family taxonomic classification. Families belonging to the same suborder are colored in the same background. The subtype proportions account for the occurrence of the *cas* gene clusters.

To explore their occurrence, we made a context analysis of the CRISPR-Cas systems in *Myxococcus* genomes. Most *Myxococcus* strains possessed the MxI-B, I-C, and III-B subtypes, but some completely sequenced genomes had the single I-C subtype only (Fig. 1A). As shown in the complete genomes, the I-C cluster had a highly similar genetic composition of almost unchanged surrounding genes in the genomes; whereas the region spanning the III-B and MxI-B gene clusters varied greatly, and an overall absence of the III-B or/and MxI-B was often observed. The residual sequences suggested that the two III-B and MxI-B subtypes had been lost independently and probably stochastically. In addition, the spacer contents in CRISPRs of the same subtypes were similar or even identical in closely related *M. xanthus* strains but greatly differed between strains with a significant evolutionary distance (Fig. S3). For example, compared to the CRISPR array in MC3.3.5c16, MC3.5.9c15 added a new unique spacer in its I-C CRISPR array, supporting that the two strains were probably separated recently (30). We infer that the CRISPR-Cas systems are inheritable components in myxobacteria with a taxonomically specific distribution pattern but are probably prone to be lost frequently.

To get a full view of myxobacterial CRISPR-Cas systems, we made a scanning in the 692 incomplete genomes and detected 473 *cas* gene clusters (Data Set S2), which, surprisingly, belonged to 19 subtypes, far exceeding eight subtypes revealed from the complete genomes. These CRISPR-Cas systems included types I, III, and IV of the class 1 system and types II and V of the class 2 system, with taxonomically distinct distributions (Fig. 1B). For example, in the *Cystobacterineae* suborder, the most popular subtype was I-C, manifested by its characteristic distribution in the family *Myxococcaceae*. For the *Nannocystineae* suborder, I-G became the most frequent subtype with the absence of MxI-B and I-C. Subtype I-G was also the feature subtype of *Sorangiineae* suborder. Among the tested myxobacterial families, *Polyangiaceae* had the most diverse CRISPR-Cas systems, containing a total of 12 subtypes. For the III-D and I-B subtypes, although their appearances were not very high, they were distributed widely in different suborders. The popular subtypes, probably depending on the genomes tested, were I-G, I-C, III-B, I-E, MxI-B, III-D, and I-B, which were all present in the complete genomes. In addition to the popular subtypes, many rare subtypes were revealed in myxobacteria, including seven hybridized systems containing *cas* genes from two different subtypes (Data Set S2). These rare subtypes mainly occurred in the *Nannocystineae*, *Sorangiineae*, and some uncertain suborders.

### Organization and phylogenetic characteristics of myxobacterial *cas* genes

**Fig 2:**
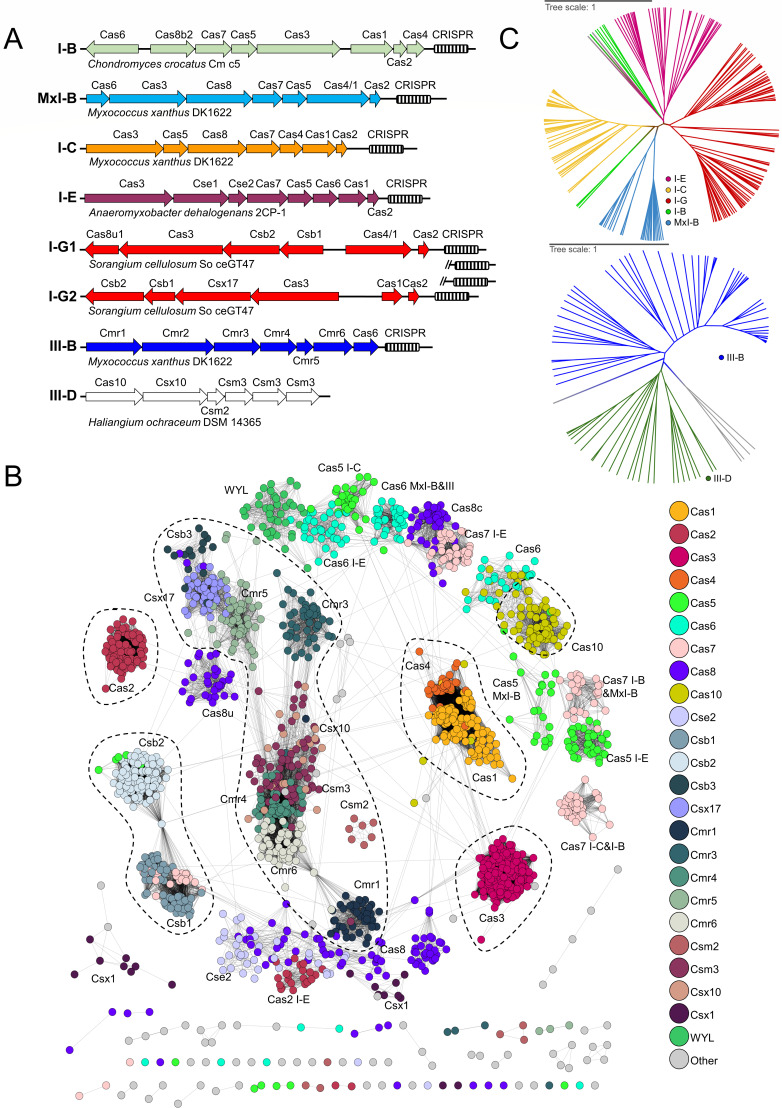
Gene organization and characteristics of the *cas* clusters in myxobacteria. (**A**) Conserved gene architectures of the seven major subtypes in representative strains with completely sequenced genomes. Note: the CRISPR array in a cluster and the gene size between different clusters are not plotted in proportion. (**B**) The similarity network of myxobacterial Cas homologs. Homologous proteins that tend to cluster together are circled. (**C**) Phylogenetic analyses of type I and type III, respectively, based on the Cas8 and Cas10 homologous proteins.

We found that among the seven popular subtypes, conserved gene compositions and arrangements were observed in MxI-B. I-C, I-G, III-B, and I-E but not in I-B and III-D. Figure 2A illustrates the *cas* organization of popular subtypes in representative strains; the *cas* organization of rare subtypes, including the hybrids, is shown in Fig. S4. MxI-B is a myxobacteria-specific subtype with the signature protein Cas8a1 (classified as I-A) or Cas8b3 (classified as I-B) (10), and the Cas1 proteins of MxI-B, fused with Cas4, are also clustered into a unique branch in the Cas1 polygenetic tree (9). In MxI-B, the Cas3-Cas8-Cas7-Cas5 cascade formed the effector module (interference module), and the upstream gene encoded the crRNA handling protein Cas6. The fused Cas1 protein, together with Cas2, constituted the adaptation module. Comparably, the *cas* gene clusters of I-C and I-E subtypes were consistent with the canonical organization (10). In I-G subtype, the interference module had four proteins: the gene pair *csb1-csb2* was followed by *cas3-cas8u1* in some cases, but followed by *cas3-csx17* in others. Notably, the two alignment models of the I-G subtype were often present in different genomes but sometimes simultaneously occurred in the same genome, like in *S. cellulosum* So ceGT47 (I-G1/I-G2 in Fig. 2A). The Csb2 protein, predicted to have the homologous domains of Cas5 and Cas6, constituted a conservative arrangement with the preceding protein Csb1. The adaptation module of I-G in myxobacteria was either not conserved, having Cas1-Cas2 or Cas4/1 (a fusion protein)-Cas2 as the adaptation module, or lacking the module. The III-B subtype had a conserved immune module arranged in the order of Cmr1-Cmr2-Cmr3-Cmr4-Cmr5-Cmr6, except that the Cmr1 unit in a few clusters was linked after Cmr3. The crRNA-processing protein Cas6 and the adaption module were often missing in the myxobacterial III-B gene cluster.

Consistent with previous reports (31, 32), the Cas1 proteins of each of the seven popular subtypes were normally highly conserved in the similarity network (Fig. 2B). The homologous proteins of Cas2 and Cas4, which are also involved in the adaptation process, similarly tended to cluster, respectively. The Cas3 homologs, responsible for the cleavage function in type I systems, also exhibited high similarity. However, the homologous proteins involved in the immune complex, such as Cas5, Cas6, Cas7, and Cas8, were phylogenetically dispersed, respectively, likely influenced by the modular origin and evolution of CRISPR-Cas systems (33). The proteins containing the WYL domain have been identified as a negative regulator of CRISPR in cyanobacteria (34) or as a positive regulator in type VI CRISPR systems (35). In myxobacteria, these WYL proteins were found in different subtypes of CRISPR-Cas but grouped in the similarity network. Notably, due to the low similarity, the Cas protein families of the I-B subtype failed to be grouped, suggesting its great diversity in myxobacteria.

The Cas proteins in the Class 1 CRISPR-Cas system were predominantly composed of single-domain subunits (except for Cas3 and the large subunits Cas8 and Cas10), but the domain classifications or Pfam architecture among homologous subunits were different (Data Set S4). We conducted phylogenetic analysis of the type I and type III systems, respectively, using their signature proteins, i.e., Cas8 for type I and Cas10 for type III. The phylogenetic trees (Fig. 2C) showed that myxobacterial subtypes were, respectively, clustered, and only the I-B subtype exhibited two branches. Although each of the subtypes was clustered, they were all deeply separated, suggesting their long evolutionary histories or quick evolution. However, motif analysis revealed that key motifs in these homologous proteins were highly conserved, such as the “HD” motif in Cas3 and the “GGDD” motif in Cas10, suggesting the functional conservation of these homologous proteins (Fig. S5 lists some key motifs that have been studied).

### Repeat-based classification and characteristics of myxobacterial CRISPR

CRISPR variation is mainly reflected in spacers, while the repeat sequences of a subtype are highly conserved and often possess conserved motifs at fixed positions (36, 37), and this characteristic could be employed to estimate the presence of CRISPR-Cas systems and the CRISPR types in fragmented genomes without relying on the *cas* cluster presence. From the 731 complete and incomplete myxobacterial genomes, we revealed 498 CRISPR arrays, which were mostly adjacent to the *cas* gene cluster with a small leader sequence. However, we noticed that the CRISPRs of I-G were not only arrayed adjacent to the *cas* genes but also scattered across the genome; they contained consistent repeat sequences, implying their origination from and service for the I-G subtype.

**Fig 3:**
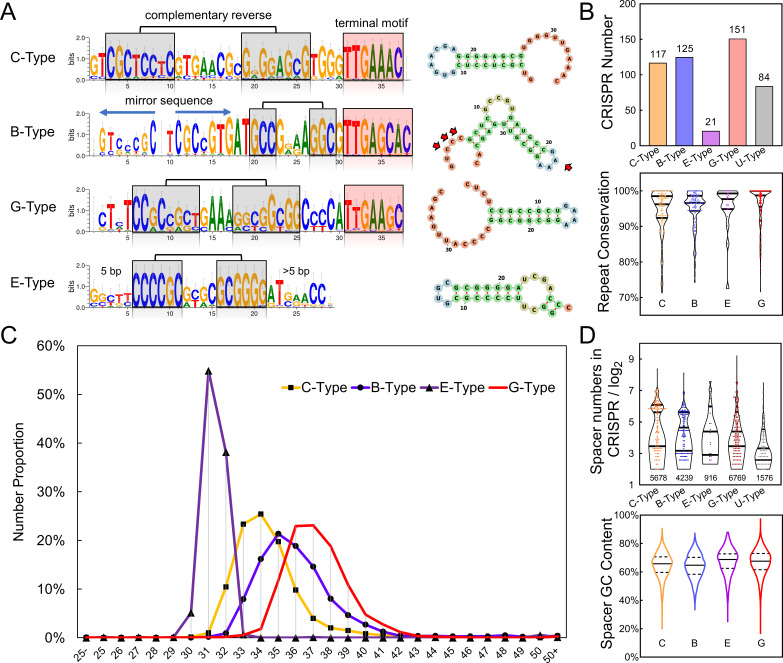
CRISPR characteristics in myxobacteria. (**A**) Four types of conserved repeat sequences and motifs of CRISPRs. The reverse palindrome sequences are marked in the gray background, and the conserved terminal motifs are labeled in pink. The sequences are shown in the 5' to 3’ orientation. Colors in the secondary structure represent different regions of repeats, and the red arrows indicate the unconservative bases in the B-Type CRISPR (Fig. S6B). The sources of representatives for repeat sequences: C-Type, I-C CRISPR of *M. xanthus* DK1622; B-Type, MxI-B CRISPR of *M. xanthus* DK1622; G-Type, I-G CRISPR of *S. cellulosum* So ce56; E-Type, I-E CRISPR of *A. dehalogenans* 2 CP-1. (**B**) CRISPR number and repeat conservation for each type of CRISPR. (**C**) Spacer length distributions for different CRISPR types. The *Y*-axis represents the number ratio of a certain length spacer to the total spacer in the CRISPR type. (**D**) Spacer number and GC content for each type of CRISPR. In the violin diagram of the spacer number, each scatter represents a CRISPR, and the number below the diagram represents the total spacer number in that CRISPR type.

Based on the repeat sequence similarity, most of the 498 CRISPRs could be classified into four types, i.e., the C-, B-, G-, and E-Types, respectively, containing 117, 125, 151, and 21 CRISPRs. The remainder (84 CRISPRs) were mostly of those rare CRISPR-Cas subtypes and were not grouped mainly due to the limited number for statistics, which we temporarily termed U-Type (unclassified type). The C- and G-Type repeats both formed the typical hairpin structure, while the E-Type and B-Type repeats had two complementary stem regions (Fig. 3A). The C-Type repeats were majorly present in the I-C CRISPRs and were 37 bp long with a conserved reverse complementary hairpin pair and the terminal motif “-TTGAAAC.” Similarly, the repeat sequences of the G-Type CRISPRs, mainly present in the I-G subtype, were 36 bp long and had the conserved tail motif “-TTGAAGC,” which was identical in either the scattered or the *cas*-adjacent CRISPRs.

The repeat sequences of the CRISPR arrays near the MxI-B and III-B gene clusters were highly similar and both possessed the conserved terminal motif “-TTGAGCAC” and a mirror sequence. For example, in *Myxococcus* strains, the MxI-B and III-B systems were adjoint in the genome, and their repeat sequences were not only in the same length (36 bp) but also highly similar (Fig. S6 demonstrates the presence of MxI-B and III-B and their repeat sequences of CRISPRs in *Myxococcus* strains), which suggested that they were likely to be alternatively employed by both MxI-B and III-B subtypes. The mismatched bases between the repeats of MxI-B and III-B subtypes appeared only in the head and loop parts of the hairpins. We tentatively grouped these CRISPRs into the B-Type for further analysis. Similar to that of B-Type, the repeats of I-E CRISPRs (E-Type) had the conserved motif “-CCCCGCNNNNGCGGGG-” characteristic and did not have a conserved tail motif.

The conservation of repeat sequences of the four CRISPR types was each above 95% (Fig. 3B). The repeat variation normally occurred in the CRISPR array away from the *cas* cluster, together with the varied old spacers. Notably, some different CRISPR-Cas subtypes might share the same repeat types. For example, the repeat sequences of the I-B, III-A, and III-D subtypes mainly belonged to the C-Type but also included some unclassified types (U-Type; refer to Data Set S2).

The spacer length reflects the uptake preference during the adaptation process and is important for subtypes to achieve immune function (38). Among the four CRISPR types in myxobacteria, the spacer length of E-Type CRISPR was very conservative, and almost all of them were at 31–32 bp in length, while the spacer sizes of C-, B-, and G-types were rather varied, and their average lengths were 34 bp, 35 bp, and 36–37 bp, respectively (Fig. 3C). The spacer number in a CRISPR array of each of the four types was greatly diverse, for example, the largest number of CRISPR spacers reached 336 in C-Type, 275 in B-Type, 191 in E-Type, and 587 in G-Type. In addition, the GC content of spacers in each myxobacterial CRISPR type was notably high, with all lower quartile values exceeding 60% (Fig. 3D), probably reflecting the preference of the adaptation module to ingest short fragments.

### Target tracing and immune history recorded in myxobacterial CRISPRs

Tracing targets for spacers can provide direct information on gene flow and potential interactions between the host and invaders (39–41). The revealed myxobacterial CRISPR arrays stored a total of 19,178 spacers, which, according to the adjoint repeat sequences, were allocated in C-Type (5,678), B-Type (4,239), E-Type (916), G-Type (6,769), and U-Type (1,576; Data Set S3). We used CRISPRTarget to search targets for these myxobacterial spacers in the databases of IMG/VR (cultured and uncultured DNA viruses and retroviruses [42]), GenBank-Phage, RefSeq-Plasmid, and RefSeq representative genomes. Of the 19,178 spacers, 13,429 obtained their targets in the four databases, accounting for 70.02%, while the other 5,749 spacers did not match any target in the databases. The number of the retrieved targets and the matching spacers are listed in Table S2. Notably, the targets of traceable spacers in different databases often overlapped to different extents (Fig. S7).

**Fig 4:**
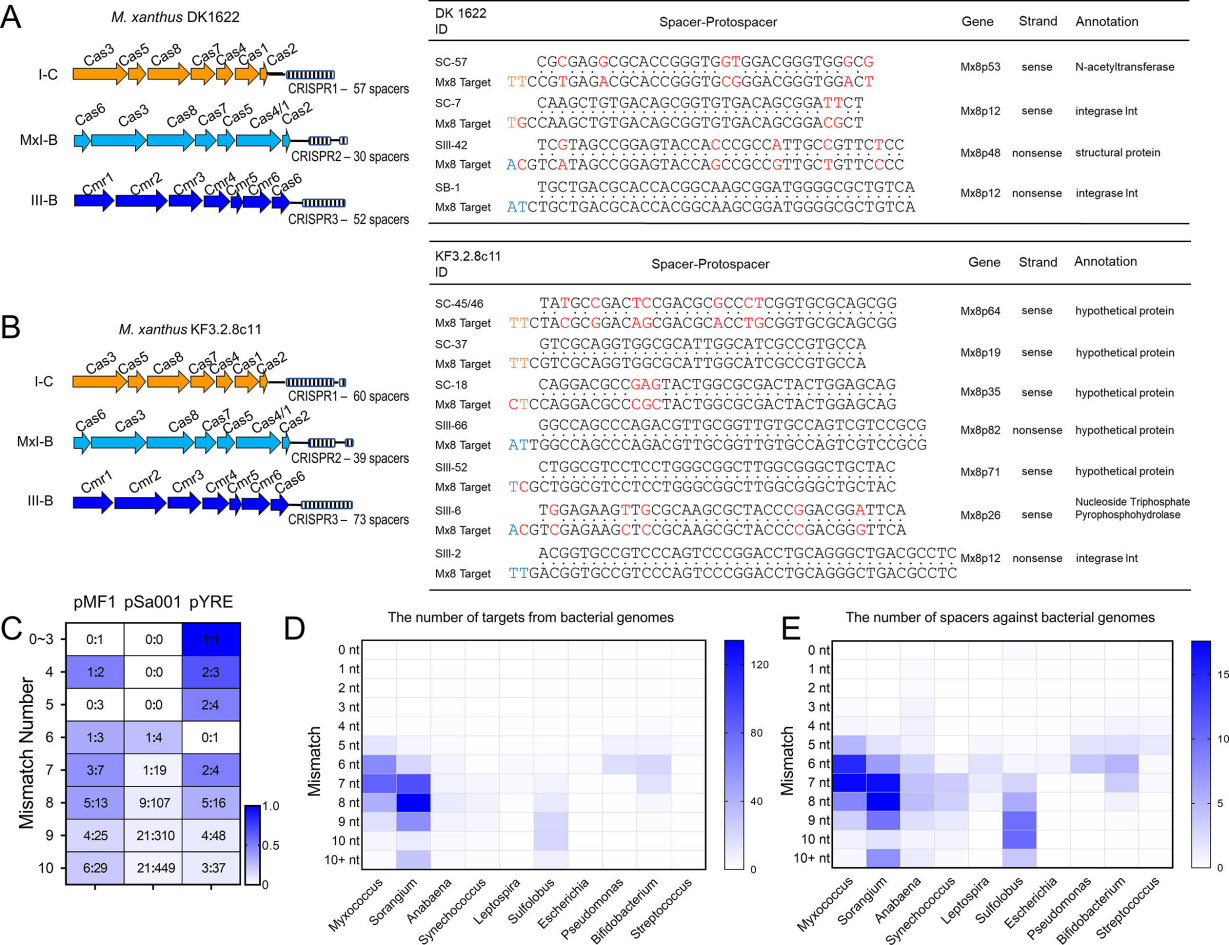
Immune histories recorded in the CRISPR arrays of myxobacteria. (**A**) The immune spacers in *M. xanthus* DK1622 against the Mx8 phage. SC-57 is the 57th spacer ID in the I-C CRISPR, and the spacers from MxI-B and III-B are similarly labeled with SB and SIII, respectively. The base mutations are marked in red, and the preferred PAM sequences for each CRISPR type refer to Fig. 5. (**B**) The immune histories recorded in *M. xanthus* KF3.2.8c11 to Mx8. SC-45/46: spacer SC-45 and SC-46 are the same. (**C**) Targets counted in myxobacterial endogenous plasmids. The PAM-containing targets to the total targets are calculated and shown before the colon in each box. Preferred PAM sequences for each CRISPR type refer to Fig. 5. (**D**) The number of targets from organismic genomes matched by the spacers from representatives of myxobacteria and other prokaryotes. (**E**) The number of spacers against organismic genomes.

In the IMG/VR and Phage databases, when the mismatches were restricted to no more than three nucleotides, the matched non-redundant targets were mostly enriched into those myxobacterial phages: 63 targeting Mx4, 78 targeting Mx8, and 13 targeting Mx9 (Fig. S8). The corresponding spacers were almost all in the C- and B-Type CRISPRs of different *Myxococcus* strains, except a few Mx4 and Mx8 targets matched by the *Corallococcus* spacers. In *M. xanthus* DK1622, there were four spacers against the Mx8 phage, two in the I-C CRISPR, and the other two, respectively, in the III-B and MxI-B CRISPRs (Fig. 4A). Among the genome-sequenced *M. xanthus* strains, KF3.2.8c11 had the largest number (seven) of Mx8-spacers in the I-C and III-B CRISPR arrays and four perfectly matched Mx8 (Fig. 4B). These results suggested that Mx8 might escape from the immune surveillance of DK1622 via the sequence mutation, but its re-invasion would again produce new spacers by different CRISPR-Cas systems. Notably, more than 35 myxophages had been isolated and identified 30–50 years ago (43), and only Mx8, Mx4, and partial Mx9 have been sequenced. We presumed that there should be more myxophages matching with *Myxococcus* CRISPR spacers. In addition, because the three *Myxococcus* myxophages matched *Myxococcus* spacers almost exclusively, there might be some unknown myxophages specifically matching other myxobacterial taxa.

Compared with the IMG/VR and Phage databases, the high-confident targets (mismatched bases ≤ 3) from the RefSeq-plasmid database were scarce, and almost no perfectly matched or high-confident target was found, even in the reported endogenous plasmids, pMF1 of *Myxococcus fulvus* 124B02 (44) and pSa001 of *Sandaracinus* sp. MSr10575 (45), both were completely sequenced along with the host genomes. If mismatched bases increased (3 < mismatched bases ≤ 10), many putative target sequences were revealed in these two endogenous plasmids (Fig. 4C). This result suggested that myxobacteria were seldom invaded by plasmids. However, different from that of myxophages, the spacers matching myxobacterial plasmids were not only present in their phylogenetically close species but also revealed in phylogenetically distant myxobacterial species (Table S3). For example, when mismatched bases ≤ 6, the spacers matching pSa001 could be revealed in the CRISPRs of *Vitiosangium*, *Anaeromyxobacter*, *Minicystis*, and *Corallococcus*. These results suggested that the pMF1 and pSa001 both had broad-spectrum hosts, and this was rather consistent with the genome composition analysis of plasmid (46).

Interestingly, by comparing the plasmid targets in myxobacteria, we discovered a third plasmid hidden in the genome of a newly isolated *Myxococcus stipitatus* strain YRE12 in our laboratory. This finding was subsequently verified by PCR amplification (Fig. S9) and complete sequencing of the genome. This circular plasmid (named pYRE) was approximately 50 kb in size and contained, compared with PMF1 and pSa001, significantly increased target-matching results (Fig. 4C).

We searched targets of the myxobacterial spacers in the RefSeq database of representative genomes using the same penalty rules of CRISPRTarget. Surprisingly, we revealed a total of 102,650 targets in the genome database, and among them, 3,697 were from myxobacterial genomes, including 91 self-matched targets (Table S2). Most myxobacterial species are typical predators and can develop complex fruiting body structures, in which an autolysis process of many vegetative cells occurs, probably for the sporulation of remaining cells (47). These processes might include an intake of nucleic acid sequences, which potentially triggered the immune response of CRISPR-Cas. The results suggested that each type of CRISPRs is functional for not only the phage or plasmid invaders but also the intakes of microbial genomes, probably during the development and predation processes.

We further analyzed the presence of spacers and their genome targets in some representatives of different taxonomic taxa, belonging to eight phyla. *Myxococcus* and *Sorangium* were selected as the representatives of myxobacteria for their more sequenced genomes. Of the comparison, *Escherichia*, *Pseudomonas*, *Bifidobacterium*, *Streptococcus*, and *Sulfolobus* were extensively studied in endogenous CRISPR-Cas systems, while *Leptospira* of *Spirochaetota*, *Anabaena*, and *Synechococcus* of *Cyanobacteria* had similar repeat sequences as those in myxobacteria. We identified CRISPRs within the genomes of these microorganisms, extracted spacers, and removed the duplicates. The unique spacers were then matched against target sequences using BLAST within the RefSeq Representative genome database. The results showed that the myxobacterium *Sorangium* exhibited the highest number of genomic targets (353 matches per strain), *Myxococcus* followed with an average of 222 matches, and these figures were significantly higher than that of other taxonomic groups (Fig. 4D). Notably, these matched targets mostly displayed more than three base pair mismatches, suggesting infrequent intakes of microbial genomes by myxobacterial cells. Similarly, the myxobacterial genera *Sorangium* and *Myxococcus* had the highest number of spacers matching genomic targets, reaching 63 and 51 unique spacers, respectively (Fig. 4E). Of the other representatives, all had more or less genome-targeting spacers, and *Sulfolobus* similarly had 34 unique spacers targeting genomes per strain (details refer to Table S4 and Data Set S5). Excluding the potentials of that carried by phages or plasmids, the results demonstrate that myxobacteria have a high tendency to uptake genomic targets.

### PAM sequences for myxobacterial CRISPR-Cas recognition

**Fig 5:**
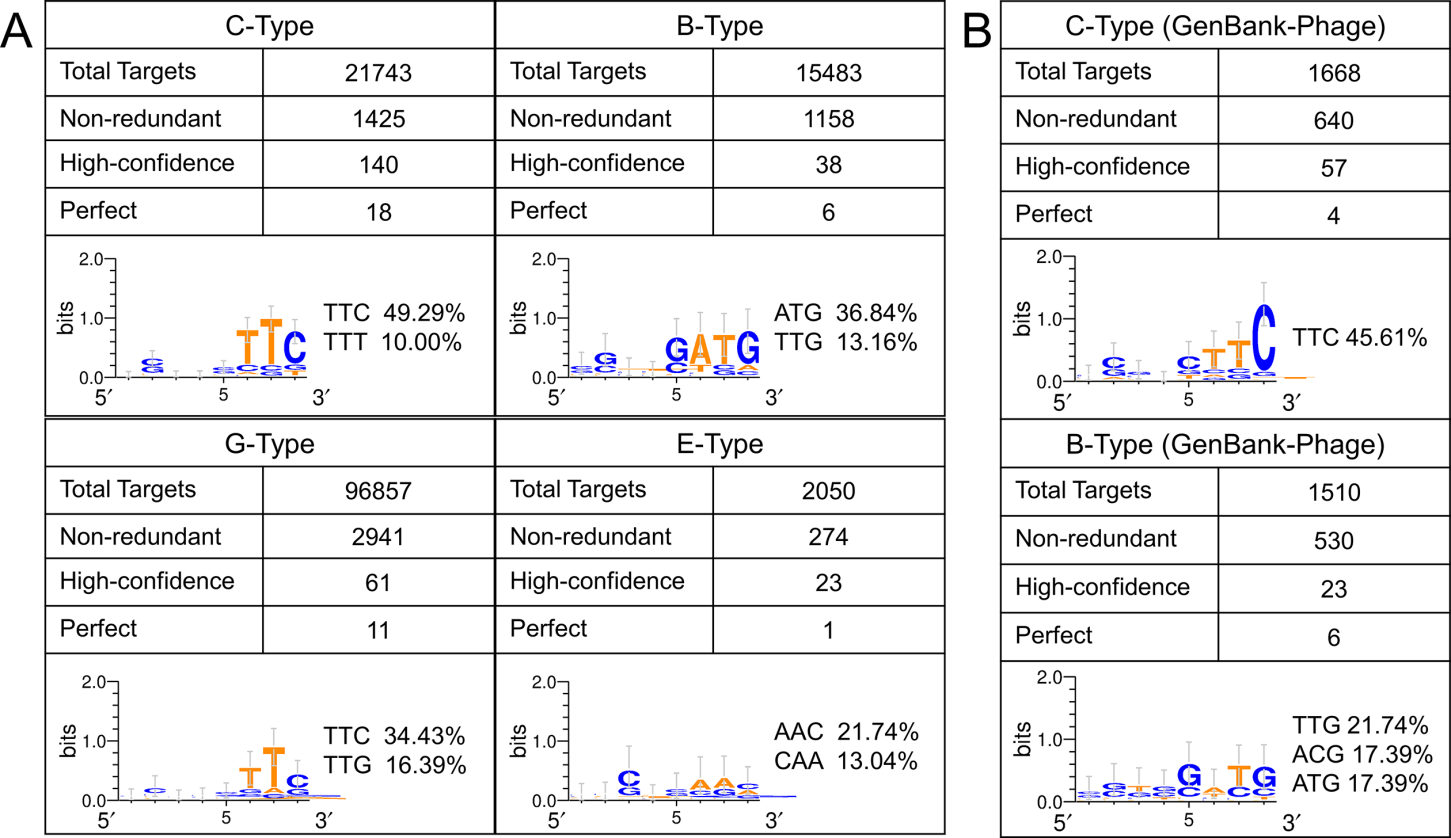
Prediction of PAM sequences in the IMG/VR (**A**) and GenBank-Phage (**B**) databases. Total target: all optimal targets matched by unique spacers for each CRISPR type. Perfect: the targets matched perfectly. High-confidence: the target contains ≤3 base mismatches with the corresponding spacer. The 5’ flanking 8 nt bases of protospacer are used for the PAM prediction. PAM sequences with a ratio of more than 10% are listed beside the conserved motif logos.

PAM is a conserved motif flanking the target sequence in intruders and is an identity marker for the recognition of some types of CRISPR-Cas systems (48, 49). Although there were massive numbers of targets in various databases for myxobacterial spacers, the number of perfectly matched targets was very small due to the incessant sequence mutation of target and spacer, as well as PAM with aging. To avoid the increasing false values caused by mismatched bases, we compared the flanking sequences of 6,222 non-redundant targets (protospacers) with different mismatches (0–6 nt; Fig. S10). Consulting the positions of PAMs in different types (50), we obtained the PAM candidates corresponding to the four types of CRISPRs [Fig. 5A demonstrates the PAMs (ratio ≥10%) of each type, and details are listed in Table S5].

For the C- and G-Types, the highest frequent PAMs were the same (5′-TTC-3′). The second most frequent PAM was 5′-TTT-3′ for the C-Type (10%) and 5′-TTG-3′ for the G-Type (16.39%). The first two preferred PAMs were 5′-ATG-3′ (36.84%) and 5′-TTG-3′ (13.16%) for the B-Type, and 5′-AAC-3′ (21.74%) and 5′-CAA-3′ (13.04%) for the E-Type. Because the third base of the PAM motif for the type I system is usually alterable (48, 51), we then counted the first two bases derived from the preferred PAMs in perfectly matched targets where mutations were most likely not to occur (Table S5). The degenerate PAMs were 5′-TTB-3′ for C-Type (88.89% frequency in perfectly matched targets; B, C/G/T), 5′-WTB-3′ for B-Type (83.33%; W, A/T), 5′-TTV-3′ for G-Type (81.82%; V, A/C/G), and 5′-AAG-3′ for E-Type (100%). All these preferred PAM types were characterized by a high AT content, contrary to the high GC content of myxobacteria genomes.

We also counted the PAM motifs with the highly matched targets of phages documented in the GenBank-Phage. Consistent with the results from the IMG/VR, the preferred C-Type PAM was 5′-TTC-3′ (45.61%), and the preferred B-Type PAM was 5′-ATG-3′ (17.39%), 5′-ACG-3′ (17.39%), or 5′-TTG-3′ (21%) (Fig. 5B; details of the potential PAM sequences are listed in Table S5). The degenerate PAMs obtained with the perfect matching targets were 5′-TTC-3′ for C-Type (100%) and 5′-WTB-3′ for B-Type (83.33%), which was consistent with the predicted result in IMG/VR. For example, the newest SB-1 spacer that perfectly matched Mx8 in *M. xanthus* DK1622 had an effective PAM of 5′-ATC-3′, and the newest SIII-2 spacer perfectly matched Mx8 in *M. xanthus* KF3.2.8c11 had an effective PAM of 5′-TTG-3′. For the three myxobacterial plasmids, the addition of preferred PAM sequences led to a sharp decrease in the target number (refer to Fig. 4C), which further suggested that myxobacteria were seldom invaded by plasmids.

### Expression and immunity of CRISPR-Cas systems in *M. xanthus* DK1622

**Fig 6:**
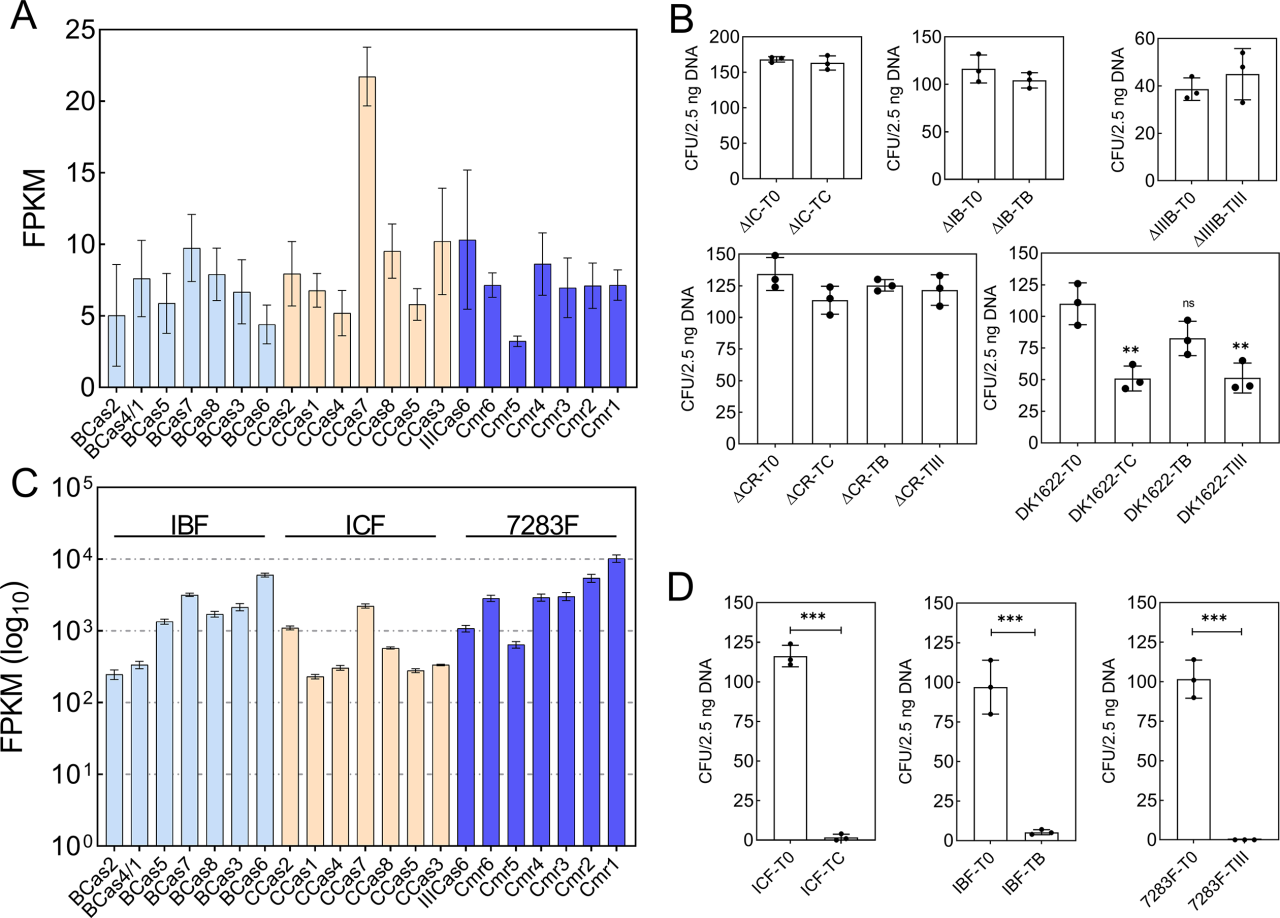
Transcription and immunity of the endogenous CRISPR-Cas in *M. xanthus* DK1622. (**A**) FPKM of the *cas* genes in DK1622 under normal culture conditions at 24 h. The RNA-seq data employed were retrieved from the NCBI accession number PRJNA760828. (**B**) Interference experiments of the endogenous CRISPR-Cas systems in *M. xanthus* DK1622 using target-equipped plasmids. Survival cells are counted after electroporation transformation, and the results are represented by the colony-forming unit (CFU). ΔIC, ΔIB, and ΔIIIB: mutants with the knockout of a single CRISPR-Cas system. ΔCR: the mutant with the knockout of all the CRISPR-Cas systems. The target-free plasmid pSWU40-T0 (T0) is the negative control, while the interference plasmids TB, TC, and TIII contain matched targets in the antibiotic resistance gene for MxI-B, I-C, and III-B, respectively. (**C**) FPKM of the *cas* genes in the promoted mutants DK1622-IBF (MxI-B promoted), DK1622-ICF (I-C promoted), and DK1622-7283F (III-B promoted). (**D**) Interference against the target-equipped plasmids by the promoted CRISPR-Cas systems. ICF: the mutant with the I-C system promoted; IBF: the mutant with the MxI-B system promoted; 7283F: the mutant with the III-B system promoted. Data in panels **B** and **D** are the means ± SD of independent experiments (*n* = 3). *P* values are calculated by Student’s *t* test to show the statistically significant differences (ns, no significance; **, *P*  <  0.01; ***, *P*  <  0.005).

Although CRISPR-Cas systems are inheritable components in myxobacteria, and many immune historic records are deposited in CRISPR arrays, there is no experimental determination of their immune functions, even in the model strain *M. xanthus* DK1622. Structural modeling and conserved motif analysis of key Cas proteins (Fig. S11) similarly suggested that the endogenous CRISPR-Cas systems in *M. xanthus* DK1622 were theoretically capable of cleaving nucleic acids. However, the RNA-seq data showed that the median FPKM (Fragments Per Kilobase of exon model per Million mapped fragments) value of DK1622 cells grown in the CTT medium for 24 hours was 26 (Fig. S12A), but almost all the *cas* genes of DK1622 had an FPKM of 10 or less (Fig. 6A). The results indicated that the endogenous CRISPR-Cas systems in DK1622 were expressed at low levels under normal growth conditions, although their transcription might be up-regulated in the developmental process (52, 53). Notably, the semi-quantitative reverse transcription PCR (RT-PCR) of the first *cas* genes and CRISPR arrays showed that the expressions of the MxI-B *cas6*, the I-C *cas3*, and the III-B *cmr1*, as well as the three CRISPR arrays, were normally very weak but surely changed at different growth stages (Fig. S12B), which might provide sufficient immune protection against genetic invaders.

To assay the immune functions of the endogenous CRISPR-Cas systems, we first constructed the deletion mutants of all three CRISPR-Cas systems (ΔCR) or a single CRISPR-Cas system (ΔIC, ΔIB, and ΔIIIB) through the traditional knockout method based on homologous recombination (54). Then, the constructed target-carrying plasmids were electroporated into *M. xanthus* DK1622 and mutant cells, respectively, to mimic the invasion of mobile genetic elements (MGEs; Fig. S13A). The interference plasmids pSWU40-TB/TC/TIII (for sequence details, refer to Table S6) were all designed with the protospacers on the noncoding strand perfectly matching the second spacers of the *cas* gene cluster-neighboring CRISPRs to ensure the possible transcription-dependent immune mechanism (29, 55, 56). The results showed that, when the target-carrying plasmids were transformed into the corresponding ΔIC, ΔIB, and ΔIIIB strains or the ΔCR strain, their transformation efficiency showed no significant difference with the target-free plasmid pSWU40-T0 (Fig. 6B). However, in the wild-type DK1622, the transformation efficiencies of plasmids carrying targets for I-C, MxI-B, or III-B were all significantly lower than that of pSWU40-T0. Therefore, the low-expressed endogenous systems in DK1622 all exhibited immune activities but not very strongly.

We further replaced the promoters with the strong constitutive promoter BBa_J23100 (57), which dramatically improved the transcription of the CRISPR-Cas clusters (Fig. 6C; Fig. S14). In the mutant of IBF (MxI-B promoted), ICF (I-C promoted), or 7283F (III-B promoted), the plasmids equipped with corresponding targets exhibited an extremely low or zero transformation rate in the same plasmid interference assay procedure (Fig. 6D). Thus, the transcriptional upregulation of the endogenous CRISPR-Cas clusters can greatly increase their immune abilities and decrease immune evasion.

### Self-target immunity and DNA deletion induced by endogenous CRISPR-Cas systems in *M. xanthus* DK1622

We designed an artificial array targeting the *pilA* gene in the genome of *M. xanthus* DK1622 to demonstrate the deletion potential with endogenous CRISPR-Cas systems. Details concerning the design and target sequences are shown in Fig. S13B. For the type-I system, the targeting spacers were designed with the repeat sequence of MxI-B (B-Type) or I-C (C-Type) to form self-target plasmids pTim-SB and pTim-SC. It is known that homology-directed repair (HDR) is the common DNA repair mechanism in myxobacteria (58). We thus further constructed self-targeting vectors containing both upstream and downstream homologous arms of approximately 800 bp, i.e., pTim-SBA and pTim-SCA, to detect whether the introduction of homologous arms could trigger the DNA repair after CRISPR-Cas degradation.

**Fig 7:**
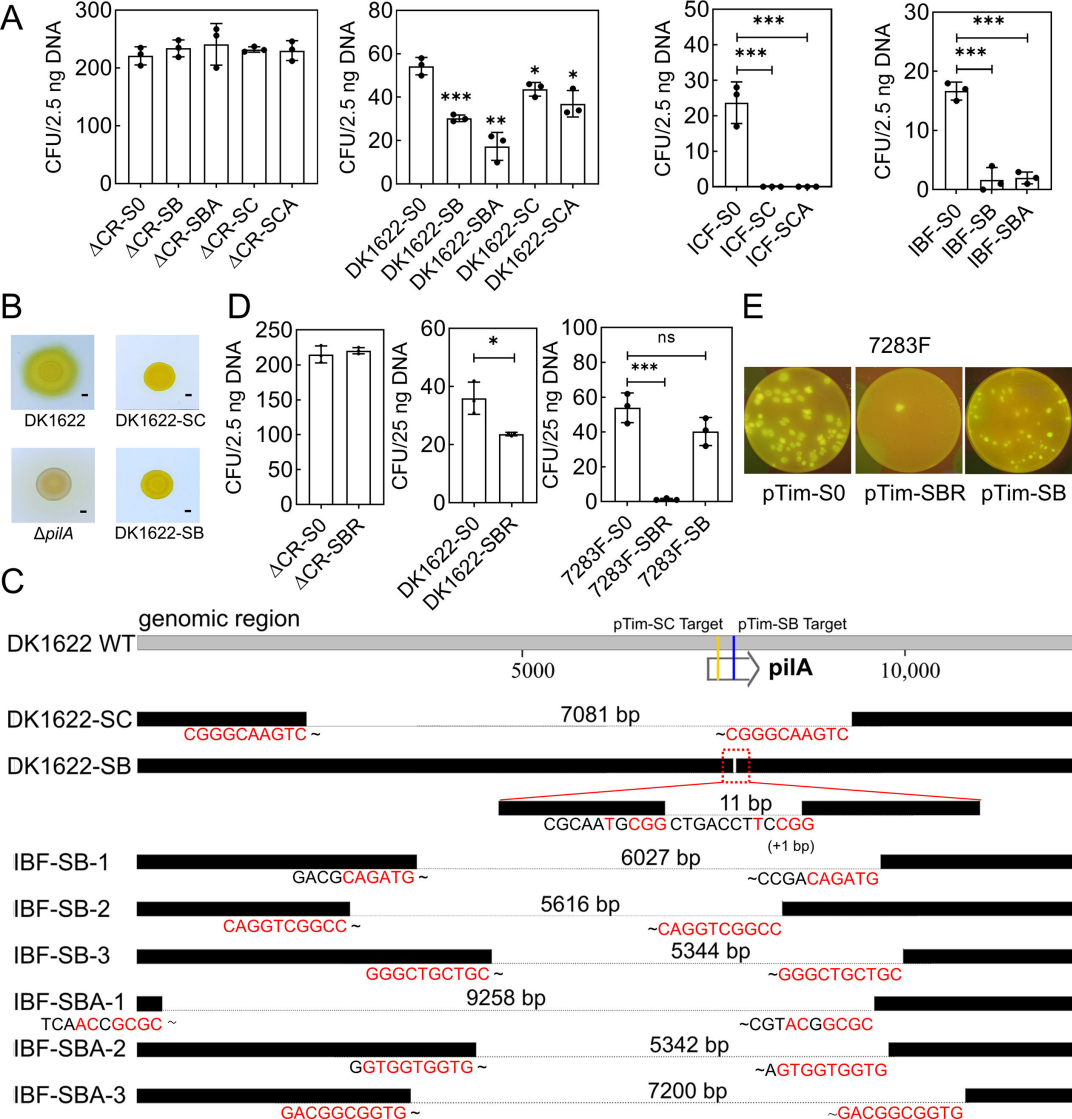
Self-target experiments of the endogenous CRISPR-Cas systems in *M. xanthus* DK1622 and its mutants. (**A**) Self-target experiments with plasmids containing artificial arrays. pTim-S0: control plasmid without array; pTim-SC and pTim-SB: self-immunity plasmids with the C- and B-Type repeat sequences, respectively; pTim-SCA and pTim-SBA: the self-immunity plasmids pTim-SC and pTim-SB, respectively, with homologous arms. (**B**) S-motility deficiency of the target deletion mutants, with the wild-type strain DK1622 and the S-motility deficient strain Δ*pilA* as reference. S-motility is on 0.4% soft agar plates, and the bar length is 2 mm. (**C**) DNA deletion and the short homologous end bases at the junctions of the deleted regions (highlighted in red). (**D**) Transformation of the self-targeting vectors pTim-SBR and pTim-SB. The spacer transcribed from pTim-SBR is designed to base pair with the RNA sequence of *pilA*, while that from pTim-SB is designed to the complementary chain of *pilA* RNA (Fig. S13B). The panel **A** and **D** data of the colony-forming unit (CFU) are the means ± SD of independent experiments (*n* = 3). *P* values were determined by Student’s *t* test to compare the samples with the control plasmid S0 (ns, no significance; *P  <  0.05; **, *P*  <  0.01; ***P  <  0.005). (**E**) The appearance of transformants from vectors pTim-S0, pTim-SBR, and pTim-SB in the III-B up-regulated strain 7283F. The reporter gene eGFP was introduced in the process of promoter replacement of CRISPR-Cas. The plates were photographed under blue light.

Compared with the target-free plasmid pTim-S0, the transformation efficiency of plasmids that carry self-targeting spacers (pTim-SB, pTim-SBA, pTim-SC, and pTim-SCA) was significantly reduced in wild-type strain DK1622 but not the ΔCR strain (Fig. 7A), which is similar to that of the plasmids targeting against exotic sequences (Fig. 6B). The results indicated that the endogenous CRISPR-Cas systems utilized introduced artificial spacers to achieve self-targeted immunity, thereby leading to a decrease in the transformation rate. Interestingly, the self-targeting vectors pTim-SBA and pTim-SCA with added homologous arms did not change the reduced transformation efficiency. This suggests that the expected HDR-based DNA repair might not have occurred. We randomly selected 48 transformants from each of the DK-SB, DK-SBA, DK-SC, and DK-SCA groups and revealed that most of the mutants did not reduce their swarming ability, except two mutants (DK1622-SB and DK1622-SC; Fig. 7B). Examining the genotypes of the S-motility-reduced mutants revealed that the target *pilA* gene was deleted at the targeting site but with different fragment ranges (Fig. 7C).

To increase the deletion efficiency, we performed the transformation of pTim-SB/SBA and pTim-SC/SCA) in MxI-B promoted (IBF) and I-C promoted (ICF), respectively, and obtained a few mutants with pTim-SB/SBA in IBF and no transformants with pTim-SC/SCA in ICF (Fig. 7A). This suggested that the promoted systems I-C and MxI-B, especially the I-C subtype, could effectively utilize artificial arrays for strong immune cleavage of genome targets, leading to a significant decrease in transformation efficiency. We randomly selected 24 colonies of each of the IBF-SB and IBF-SBA mutants and revealed that approximately 1/4 of these colonies (6/24 in IBF-SB and 5/24 in IBF-SBA) were deficient in S-motility. Three of each of the IBF-SB and the IBF-SBA were sequenced of the changes at the *pilA*-targeting site. Surprisingly, all these six mutants were deficient at the targeting site but with varied deletion fragments. Thus, the self-immunity triggered by the endogenous CRISPR-Cas system might lead to random deletion of DNA segments covering the target sites. Analyzing the deletion sites showed the presence of short homologous end bases at the junctions of the deleted regions (Fig. 7C; Fig. S15). This interesting finding suggested that the mutants with target deletions in IBF-SB and IBF-SBA were not repaired via HDR. Instead, the MMEJ mechanism (59, 60) might be involved in the DNA repair for the DNA cleavage triggered by the self-immunity of the MxI-B CRISPR-Cas system, which has not been reported in myxobacteria. Because DK1622-SC similarly had short homologous end bases at the junctions of the deleted regions, we inferred that the MMEJ mechanism was also involved in the I-C DNA cleavage.

As for III-B, the plasmid pTim-SBR was designed to equip the protospacer on the noncoding strand to assess the self-immunity capability (for design details, refer to Fig. S13B). The plasmids pTim-SBR exhibited a significant decrease of transformation efficiency in the wild-type DK1622 but not the strain deficient of the endogenous CRISPR-Cas systems (ΔCR). The MxI-B possesses almost the same repeat sequences as III-B, thus probably influencing the immunity conclusion of III-B. We further constructed the plasmid pTim-SB equipped with the protospacer on the coding strand and transferred the pTim-SBR and pTim-SB plasmids, as well as the control plasmid pTim-S0, respectively, to the III-B-promoted mutant strain 7283F. Similarly, the immunity efficiency of pTim-SBR was greatly improved, while pTim-SB had almost no difference with pTim-S0 (Fig. 7D and E), confirming the transcription-dependent immune characteristics of the III-B system. By analyzing the relative proportions of pTim-SBR in DK1622 and the III-B-promoted mutant strain, we inferred that the endogenous III-B system utilizes the B-type CRISPR array for self-immunity. We checked the phenotypes of some DK1622-SBR or 7283F-SBR transformants, which, however, did not reveal S-motility deficient mutants. We thus did not know whether the MMEJ mechanism was involved in the III-B DNA cleavage.

Notably, we also selected some mutants with normal S-motility and sequenced the changes at the *pilA*-targeting site. Some of these immune escapers were determined to have no change at the targeting site but lose the artificial array in the plasmid, and some had no changes in either the array or target sequences, with unknown reasons for their escape, requiring further investigations.

## DISCUSSION

The essential function of CRISPR-Cas systems is to destroy the recognized genetic materials, thus serving as an adaptive immune system for the host against invaders. Endogenous CRISPR-Cas systems have been bioinformatically analyzed at different taxonomic levels of some prokaryotes, e.g., in the *Streptococcus mutans* strain P42S (61), among different isolates of *Proteus mirabilis* (62), within the genus of *Bifidobacterium* (50), the family of *Mycobacteriaceae* (63), the phylum of cyanobacteria (64), or even the whole prokaryotes (10). Recent advances in sequencing and bioinformatic platforms have energized the assembly of prokaryotic genomes (65, 66). Besides the well-sequenced strains, massive incomplete genomes, including MAGs from environmental DNAs, are also indispensable for the collation and analysis of CRISPR-Cas systems, especially in those taxonomic groups with limited complete genomes. Although metagenomic sequencing and splicing may help us skip cultivation to excavate the CRISPR-Cas systems from uncultivated microbes (67), the possible subtype diversity may pose a challenge for data collation, but the analysis with lots of incomplete genomes, including MAGs binned from environments, will undoubtedly make the result more inclusive and comprehensive. The analysis route in this study provides a methodological reference for a comprehensive understanding of CRISPR-Cas in a specific microbial group.

Using the combined pipeline, our analysis of 39 complete genomes and 692 incomplete genomes revealed that myxobacteria possess at least 19 subtypes of the CRISPR-Cas systems, which far exceed the subtypes observed in the complete genomes of myxobacteria collected in the CRISPRCasdb database (28). The seven major subtypes (I-B, I-C, MxI-B, I-E, I-G, III-B, and III-D) were often taxonomically distributed in myxobacteria, while the rare subtypes were mostly detected in unclassified myxobacteria in *Nannocystineae* and *Sorangiineae*, as well as those in uncultured suborders. Because there are a large number of uncultured myxobacteria (68), more CRISPR-Cas systems are expected to be present in those unexplored myxobacteria. The inheritance of CRISPR and the phylogenetic analysis of signature Cas proteins showed that myxobacterial CRISPR-Cas systems are probably inherited from the same ancestors, probably through MGEs (69). Some popular subtypes are supposed to enter the ancestral myxobacterial cells at earlier evolutionary stages since they are widely distributed with conserved *cas* and CRISPR characteristics. However, these CRISPR-Cas systems may be defective or lost in strains, probably in the process of balancing ecological benefits (7, 70).

In myxobacteria, traceable targets of CRISPR spacers appeared in limited myxobacteria-related MGEs (two phages Mx4 and Mx8, and two endogenous plasmids pMF1 and pSa001) but frequently in unknown viruses. Relative to a large number of anti-phage immune traces, plasmid targets are rare, which may indicate that fewer plasmids can be stably transferred among myxobacteria. We also found numerous highly matched targets derived from microbial genomes, and this strongly suggested that CRISPR-Cas in myxobacteria may play an important function beyond adaptive immunity. In addition, we also observed some self-target sequences from their own genomes. However, because matched spacers were mostly with more than three base mismatches, targeting microbial genomes, including self genomes, occurred infrequently, or spacer sequences were mutated frequently. We demonstrated that the target located within the genome of *M. xanthus* DK1622 is rather lethal when the endogenous CRISPR-Cas system is highly transcribed (Fig. 7). Further investigations are required to understand the numerous spacers derived from microbial genomes, as well as the own genomes.

*M. xanthus* DK1622 possesses three endogenous CRISPR-Cas systems. The MxI-B subtype in DK1622 was initially reported as the *dev* operon functioning in cell development (21, 71), while the III-B subtype functioned in the production of extracellular polysaccharides (23) and was regulated by an extracytoplasmic function σ/anti-σ pair (24). In addition, the deletion of MxI-B *cas* genes along with the upstream genes *MXAN_7266* and *MXAN_7267* resulted in the loss of predatory ability of *M. xanthus* cells against *Bacillus subtilis* (72). However, it is not known whether the above physiological phenotypes related to the CRISPR-Cas genetic loci were associated with the immune functions of the endogenous CRISPR-Cas systems, which also require more investigations. Despite these “non-immune” functions, the immunity of these endogenous CRISPR-Cas systems in *Myxococcus* has not been mensurated before. Here, we showed that I-C, MxI-B, and III-B in DK1622 all have low expression levels when cells grow normally, but they do resist the invasion of plasmid and provide stronger resistance when their transcripts are uplifted by promoter replacement. Notably, an increase in transcription of endogenous CRISPR-Cas systems was observed during development (52, 53), and some unknown invasion signal may trigger the ECF-σ/anti-σ pair of III-B (24). The increased transcription in some specific living stages might strengthen the immune functions of the endogenous CRISPR-Cas system.

Unexpectedly, the DNA repair resulting from immune cleavage by the endogenous CRISPR-Cas systems seems not to involve the common HDR mechanism in *M. xanthus* (58) but rather the MMEJ DNA repair mechanism. It has been reported that HDR and MMEJ repair pathways might compete with each other (60). When conditions favor HDR, such as increasing the number of homologous arm copies, precise repair mediated by homologous arms can be achieved, even suppressing MMEJ (59). MMEJ has not been reported in myxobacteria but has been documented in *E. coli* (60), *P. aeruginosa* (60), and *Zymomonas mobilis* (59), as well as an archaeal organism (73). In DK1622, the CRISPR-Cas-induced MMEJ results in random loss of DNA fragments spanning the target site, ranging from 4 to 9 Kb, probably being a potential genetic tool in myxobacteria, and also requiring deep studies for understanding HDR and MMEJ repair pathways, as well as their relationships in *M. xanthus*. In conclusion, the endogenous CRISPR-Cas of myxobacteria is an excellent research object and mining resource for further exploration.

## MATERIALS AND METHODS

### Collection of myxobacterial genomes

All the myxobacterial genomes available in the NCBI Genome database (up to 5 January 2022) were retrieved (Data Set S1). There is a total of 731 myxobacterial genomes (six anomalous assemblies were excluded), of which 39 were completely sequenced, and the others were either incompletely sequenced and released in scaffolds and contigs or assembled from metagenome sequences. The genome assemblies of uncultured myxobacteria to be analyzed were downloaded from Ref-seq if they existed, otherwise were obtained from GenBank. Notably, although the taxonomy of myxobacteria has been reclassified as the *Myxococcota* phylum in 2020 (19), this paper uses the classification system of the *Myxococcales* order annotated by GenBank at the time.

### CRISPR-Cas mining and classification

The myxobacterial genomes were submitted to the CRISPRCasTyper server (74) to detect the CRISPR and Cas components. The clusters possessing several *cas* genes were subtyped mainly according to the signature Cas proteins (10), and the annotations of other Cas proteins were employed as references if necessary. The CRISPR sequences were redetected using the online tools CRISPRCasFidner (75), and the CRISPR arrays with the evidence level = 4 were extracted for further analysis. In some incomplete genomes, a few CRISPRs containing no neighboring *cas* clusters, thus could not be subtyped using *cas*, were tentatively classified according to the CRISPR types.

### Phylogenetic tree construction

Software Prodigal was used for the protein annotation of the genome, and then the PhyloPhlAn program (76) was used to construct phylogenomic trees (parameter settings: -p meta -q, others default). For molecular evolution analysis of Cas proteins and repeat sequences, we used the software MAGE11 with the method of neighbor-joining and parameter default. All the phylogenetic results were drawn using the online software package ggtree (77) or iTol (78).

### CRISPR-Cas genetic context analysis

For the incomplete genomes lacking complete CRISPR-Cas information, the genes near the CRISPR-Cas cluster of the phylogenetically close strains with completely sequenced genomes were employed to locate possible positions of the lacked CRISPR-Cas genes. Briefly, appropriate genes before and after a CRISPR-Cas cluster were retrieved with the location information. The proteins encoded by these CRISPR-Cas surrounding genes in a genome were collected as a library, and the libraries of phylogenetically close myxobacteria were compared for their sequence similarities by using BLAST+ with the output parameter of “-outfmt 6” and the other parameters of default. After filtering (excluding subject sequences with e-value > 1 or pident < 70), the genetic structures were plotted using genoPlotR (79). The extracted CRISPRs were visualized using CRISPRstudio (80).

### Trimming and analysis of the Cas proteins

For reconfirmation, the Cas proteins annotated by CRISPRCasTyper (CCTyper) were compared with the sequences recorded in the NCBI database and then were submitted to the EFI Web Resource (81) to analyze the sequence similarity network with default parameters. The score threshold was set as one, and the nodes with 90% identity were integrated as one. Groups were manually circled according to the subtype annotation.

The Cas protein sequences were submitted to the Pfam search online program to retrieve the matched Pfam families against the HMMER database. The Multiple Em for Motif Elicitation program was used to search for conserved residues and motifs in each group of homologous proteins.

### Target tracing and PAM prediction

The CRISPR spacers were traced using the software CRISPRTarget (82) against the IMG/VR, GenBank-phage, and RefSeq-plasmid databases. The parameters of the penalty rule were set as default, and the target score threshold was set to 20. The eight nucleotides (nt) flanking the protospacers were extracted for the prediction of the PAM sequences, which were then summarized in different target sets. Since no prokaryotic genome database is selected by CRISPRTarget, we used BLAST to trace targets against the representative genomes in the RefSeq genome database with the same penalty rule as that in CRISPRTarget.

### Strains and cultural conditions

The strains used in this study are listed in Table S7. Myxobacterial strains were routinely cultivated in the CTT liquid medium (1% casitone, 8 mM MgSO_4_, 10 mM Tris-HCl, 1 mM K_2_HPO_4_/KH_2_PO_4_, and pH 7.6) (83) at 30°C. The *E. coli* strains were grown in the lysogeny broth (LB) medium at 37°C. Agar (1.5%) was added to prepare the corresponding solid media. To screen transformants, 40 µg/mL kanamycin or 30 µg/mL apramycin were added as the resistance required.

### Promoter replacement

To up-regulate the transcription of endogenous CRISPR-Cas in *M. xanthus* DK1622, the native promoters were replaced with a composite promoter of the core promoter BBa_J23100 and the ribosome-binding site BBa_B0034 using the single homologous arm recombination method, and the detailed method was reported in reference 57. The chosen promoter was added before the first genes of MxI-B (*MXAN_RS35160* [BCas6]), I-C (*MXAN_RS33980* [CCas3]), and III-B (*MXAN_RS37245* [*MXAN_7283*]), respectively.

### Transcription determination

The DK1622 cells grown in CTT for 24 hours were collected for the extraction of RNA. After the removal of DNA, the total RNA was reversely transcribed into cDNA using the reagent “HiScript III RT SuperMix for quantitative PCR” (qPCR; Vazyme Company, Nanjing, China). Semi-quantitative RT-PCR was used to detect the CRISPR-Cas transcription. Real-time qPCR analysis was performed on the instrument qTOWER3 G IVD using the SYBR Green I. The relative transcriptional levels in mutants relative to those in the wild type were analyzed using the 2^(-ΔΔCt)^ method. The primers used are listed in Table S8. The method of transcriptional sequencing was recorded in the reference (57).

The detailed procedure of RNA-seq was documented in the previous work (57). Briefly, the CRISPR-Cas activated mutants and the wild-type DK1622 were, respectively, inoculated in the liquid CTT and cultivated to the stationary phase. The cultures were then transferred to a fresh antibiotic-free medium with the same initial OD_600_ values in triplicate for each strain. After 24 hours of incubation, cells were collected and stored at −80°C before transcriptome sequencing, which was conducted in Novogene Biotech (Beijing, China) using an Illumina Hiseq 4000 platform.

### PCR verification of the presence of the pYRE plasmid

To verify the presence of pYRE, we amplified fragments of the 50 Kb plasmid using the total DNA as the template. The DNA was extracted as previously described (44). The plasmid was divided into nine segments, and the homologous arms (~500 bp) were designed between every two fragments. To increase the cloning efficiency of long fragments from high-GC genomes, 1/10 volume of dimethyl sulfoxide was added to the PCR mixture (84). The PCR fragments were completely reconfirmed using Sanger Sequencing, and the complete pYRE plasmid sequence was uploaded to NCBI with the accession ID of CP102771.

### Assay for immunity by plasmid interference

To construct interference plasmids, the pSWU30 plasmid (85), which has a perfect MxI-B matching target (5′-TGCTGACGCACCACGGCAAGCGGATGGGGCGCTGTCA-3′), was used as the starting vector for the plasmid interference experiment in *M. xanthus* DK1622. The *tet^R^* in pSWU30 for tetracycline screening was replaced with the apramycin resistance gene (*apr^R^*). Subsequently, the target gene was mutated using a codon senseless mutation (the mutation sequence is shown in the corresponding primers listed in Table S8) to obtain the target-free plasmid pSWU40-T0. As for the interference plasmids, the protospacers with the predicted PAM sequence “TTC” (for I-C), “ATG” (for MxI-B), and “CCC” (for III-B) were, respectively, designed in the 5' untranslated region of the apramycin resistance gene (*apr^R^*) on the noncoding strand.

The recipient strains were cultivated overnight to the exponential growth phase, then an appropriate amount was taken and centrifuged. The cells were washed three times with 10% glycerol, resuspended in 10% glycerol to an OD_600_, and stored in aliquots at −80°C. For electroporation, the stored competent cells were thawed, added with 100 ng plasmid, mixed softly and thoroughly, and then subjected to electroporation under the condition of 625 V/mm, 25 µF, and 400 Ω. The electroporated cells were recovered in 2 mL of antibiotic-free CTT liquid medium for 4 hours, followed by the addition of the appropriate antibiotic and incubation for 2 additional hours. The cell suspensions were mixed with CTT medium containing 0.4% agar, and different dilutions were spread on solid agar plates. After 5–7 days of incubation, the transformants were counted, and suitable colonies were picked for subsequent experiments.

### Plasmid construction for self-immunity by CRISPR-Cas systems

The pMF1 ori (44) was used for the construction of self-replication plasmid pTim-S in *M. xanthus*. The self-targeting sites were selected from the *pilA* gene (*MXAN_RS28035*) with the corresponding PAM. The selected protospacers were designed between the B- and C-Type repeat sequences in DK1622. The artificial array expression cassette promoted by a constitutive promoter pSH058 (57) was integrated into the pTim-S vector to form self-targeting vectors. The target-free vector pTim-S0 was used as the control. The plasmids and primers are listed in Tables S7 and S8.

## Data Availability

The *Myxococcus stipitatus YRE12* genome and its plasmid pYRE sequence were deposited in the GenBank database with the accession numbers CP102770 and CP102771. The raw RNA-seq data of the CRISPR-Cas promoted strains were uploaded to NCBI with the BioProject as PRJNA917052.

## SUPPLEMENTAL MATERIAL

Data Set S1 msystems.01210-23-s0001.xlsx Collection of myxobacterial genomes.

Data Set S2 msystems.01210-23-s0002.xlsx CRISPR-Cas in myxobacteria.

Data Set S3 msystems.01210-23-s0003.xlsx Spacers recorded in the CRISPR arrays of myxobacteria.

Data Set S4 msystems.01210-23-s0004.xlsx Pfam architectures of *cas* homologs.

Data Set S5 msystems.01210-23-s0005.xlsx Genomic targets in myxobacterial representatives and other representative taxa.

Supporting information msystems.01210-23-s0006.pdf Tables S1-S8 and Figures S1-S15.

ASM does not own the copyrights to Supplemental Material that may be linked to, or accessed through, an article. The authors have granted ASM a non-exclusive, world-wide license to publish the Supplemental Material files. Please contact the corresponding author directly for reuse.
