# Supplementary material for: Characteristics and immune functions of the endogenous CRISPR-Cas systems in myxobacteria
Source: mSystems. 2024 May 15;9(6):e01210-23. doi: 10.1128/msystems.01210-23 (PMC11237760; doi:10.1128/msystems.01210-23)
Supplement: Supporting information — Tables S1-S8 and Figures S1-S15. [file msystems.01210-23-s0006.pdf]

Supporting information for the manuscript:

Characteristics and immune functions of endogenous CRISPR-Cas systems in the social and predatory myxobacteria

Wei-feng Hu[1], Jiang-yu Yang[1], Jing-jing Wang[1], Shu-fei Yuan[1], Xin-jing Yue[1], Zheng Zhang[1], Ya-qi Zhang[1], Jun-yan Meng[1], Yue-zhong Li[1]*

[1]State Key Laboratory of Microbial Technology, Institute of Microbial Technology, Shandong University, 266237 Qingdao, P.R. China

*** The corresponding author:** Yue-zhong Li, lilab@sdu.edu.cn; ORCID ID, 0000-0001-8336-6638.

Content:

Supplementary Table 1. Taxonomic statistics of sequenced myxobacteria.

Supplementary Table 2. Traced targets for CRISPR spacers of myxobacteria.

Supplementary Table 3. Traced targets in endogenous plasmids of myxobacteria.

Supplementary Table 4. A comparison of the genomic targets and genome-targeting spacers in myxobacterial representatives and other representative taxa.

Supplementary Table 5. PAM Candidates and the suggested preferred PAMs for each CRISPR type.

Supplementary Table 6. The target design for plasmid interference assays.

Supplementary Table 7. Strains and plasmids used in this study.

Supplementary Table 8. Primers used in this study.

Supplementary Figure 1. The pipeline for the analysis of endogenous CRISPR-Cas systems in myxobacteria with a few complete genomes and lots of incomplete genomes.

Supplementary Figure 2. The CRISPR-Cas systems retrieved in myxobacterial complete genomes.

Supplementary Figure 3. Spacer contents in the CRISPR arrays of different *Myxococcus xanthus* strains having complete genomes.

Supplementary Figure 4. Endogenous CRISPR-Cas systems in myxobacteria beyond the seven major subtypes (shown in **Figure 2A**).

Supplementary Figure 5. Critical residues and motifs of the homologues in every Cas protein.

Supplementary Figure 6. B-Type CRISPRs in MxI-B and III-B subtypes.

Supplementary Figure 7. Statistics of the traceable spacers in four databases.

Supplementary Figure 8. Myxobacterial spacers targeting against phages.

Supplementary Figure 9. PCR verification of the pYRE plasmid.

Supplementary Figure 10. PAM determination in non-redundant targets derived from IMG/VR.

Supplementary Figure 11. Determination of key motifs for DNA and RNA handling Cas proteins.

Supplementary Figure 12. Transcription of CRISPR-Cas.

Supplementary Figure 13. Designs for function verification of CRISPR-Cas**.**

Supplementary Figure 14. Endogenous CRISPR-Cas activation by replacing the native promoters.

Supplementary Figure 15. Identification of target deletion fragments induced by CRISPR-Cas self-immunity.

Supplementary Table 1. Taxonomic statistics of sequenced myxobacteria.

Order	Suborder	Family	Genus	Species	Genome Number
Myxococcales	*Cystobacterineae*	*Anaeromyxobacteraceae*	*Anaeromyxobacter*	A. *dehalogenans*	6
				unclassified *Anaeromyxobacter* spp.	22
			unclassified spp. of *Anaeromyxobacteraceae*		2
		Archangiaceae	*Archangium*	A. *gephyra*	3
				A. primigenium	1
				A. violaceum	3
				unclassified *Archangium* spp.	8
			Cystobacter	*C. ferrugineus*	1
				C. fuscus	2
				C. gracilis	1
				unclassified *Cystobacter* spp.	1
			Hyalangium	*H. minutum*	1
				unclassified *Hyalangium* spp.	1
			Melittangium	*M. boletus*	1
			Stigmatella	*S. aurantiaca*	3
				S. erecta	1
				S. hybrida	1
			Vitiosangium	unclassified *Vitiosangium* spp.	1
			unclassified spp. of *Archangiaceae*		2
		Myxococcaceae	*Aggregicoccus*	unclassified *Aggregicoccus* spp.	1
			Citreicoccus	*C. inhibens*	1

			Corallococcus	*C. aberystwythensis*	1
				C. carmarthensis	2
				C. coralloides	3
				C. exercitus	3
				C. exiguus	13
				C. interemptor	1
				C. llansteffanensis	1
				C. macrosporus	3
				C. praedator	1
				C. sicarius	1
				C. terminator	1
				unclassified *Corallococcus* spp.	26
			Myxococcus	*M. eversor*	1
				M. fulvus	2
				M. hansupus	1
				M. llanfair	1
				M. stipitatus	1
				M. vastator	1
				M. virescens	2
				M. xanthus	18
				unclassified *Myxococcus* spp.	23
			Pyxidicoccus	*P. caerfyrddinensis*	1
				P. fallax	2
				P. trucidator	1
				unclassified *Pyxidicoccus* spp.	1

			Simulacricoccus	unclassified *Simulacricoccus* spp.	1
			Myxococcaceae incertae sedis	*Candidatus* Fimimonas gallinarum	1
				Candidatus Fimimonas merdipullorum	1
			unclassified spp. of *Myxococcaceae*		24
		Vulgatibacteraceae	*Vulgatibacter*	*V. incomptus*	1
		unclassified spp. of *Cystobacterineae*			4
	Nannocystineae	*Kofleriaceae*	*Haliangium*	*H. ochraceum*	3
				unclassified *Haliangium* spp.	1
			Unclassified spp. of *Kofleriaceae*		16
		Nannocystaceae	*Nannocystis*	*N. exedens*	4
				N. pusilla	1
				unclassified *Nannocystis* spp.	4
			Plesiocystis	*P. pacifica*	1
			Pseudenhygromyxa	unclassified *Pseudenhygromyxa* spp.	1
			unclassified spp. of *Nannocystaceae*		5
		Unclassified spp. of *Nannocystineae*			3
	Sorangiineae	*Labilitrichaceae*	*Labilithrix*	*L. luteola*	1
				unclassified *Labilithrix* spp.	7
		Polyangiaceae	*Byssovorax*	*B. cruenta*	1
			Chondromyces	*C. apiculatus*	1

				C. crocatus	1
			Pajaroellobacter	*P. abortibovis*	1
			Polyangium	*P. aurulentum*	1
				P. fumosum	1
				P. spumosum	1
			Sorangium	*S. cellulosum*	16
				unclassified *Sorangium* spp.	2
			unclassified spp. of *Polyangiaceae*		31
		Sandaracinaceae	*Sandaracinus*	*S. amylolyticus*	1
				unclassified *Sandaracinus* spp.	16
			unclassified spp. of *Sandaracinaceae*		18
	Sorangiineae incertae sedis		*Minicystis*	*M. rosea*	1
				unclassified *Minicystis* spp.	1
		unclassified spp. of *Sorangiineae*			1
Myxococcales incertae sedis			*Enhygromyxa*	*E. salina*	3
unclassified spp. of *Myxococcales*					381
Total					**731**

Supplementary Table 2. Traced targets for CRISPR spacers of myxobacteria. The strategy for target tracing is described in the method section, and the matched sequences with a score of more than 20 are regarded as potential targets and are counted. Targets revealed some from self or other myxobacterial genomes are shown in columns with a grey background. The repetitions of the targets matching the same spacer are removed before counting.

Type	Targets	Targets in IMG/VR		Targets in GenBank-Phage		Targets in Refseq-Plasmid		Targets in Representative genomes		Targets of myxobacteria		Self-matched targets	
C	57465	21743	37.84%	1685	2.93%	12609	21.94%	21428	37.29%	1305	2.27%	13	0.02%
B	45781	15483	33.82%	1493	3.26%	10125	22.12%	18680	40.80%	1162	2.54%	20	0.04%
E	10105	2050	20.29%	388	3.84%	3583	35.46%	4084	40.42%	65	0.64%	0	0.00%
G	196805	96857	49.21%	5568	2.83%	38394	19.51%	55986	28.45%	1105	0.56%	58	0.03%
U	14011	7557	53.94%	514	3.67%	3468	24.75%	2472	17.64%	60	0.43%	0	0.00%
Total	331496	143690		9648		68179		102650		3697		91	

Type	Spacers	Spacers against IMG/VR		Spacers against GenBank-Phage		Spacers against Refseq-Plasmid		Spacers against Representative genomes		Spacers against myxobacteria		Spacers against self-genome	
C	5678	1957	34.47%	933	16.43%	1878	33.08%	2843	50.07%	604	10.64%	11	0.19%
B	4239	1717	40.50%	770	18.16%	1479	34.89%	2358	55.63%	455	10.73%	18	0.42%
E	916	303	33.08%	170	18.56%	364	39.74%	483	52.73%	53	5.79%	0	0.00%
G	6769	3434	50.73%	1496	22.10%	3250	48.01%	4452	65.77%	702	10.37%	18	0.27%
U	1576	476	30.20%	207	13.13%	461	29.25%	354	22.46%	30	1.90%	0	0.00%
Total	19178	7887		3576		7432		10490		1844		47	

Supplementary Table 3. Traced targets in endogenous plasmids of myxobacteria. The targets with ≤ 6 mismatches are listed.

Spacer ID	Spacer Host	Target Sequence	Score	Target Resource	Mismatch
SC2124	*Myxococcus* sp. AB025B	GCGAGAGACTGAGTCCCGGCCTTAGGGGCTGAGC	30	pMF1	2
SB682	*Myxococcus xanthus* strain KF3.2.8c11	ATGAGCAGAGGTTGCAGCTCGTGCTTCGTGCGCCG	27	pMF1	4
SC189	*Myxococcus hansupus* strain contaminant ex DSM 436	GGGTCGAGTGGCCGTCGAGCCATGTCGAGGCG	24	pMF1	4
SB2376	*Myxococcus* sp. AB056	GTCCAGGGTTGGACGAACGCTTGTTAGAGGCCCTG	23	pMF1	6
SC2113	*Myxococcus* sp. AB025B	CCGAAGGGCACGTGCCCGGTACCCGGCCAGAC	20	pMF1	6
SC3275	*Myxococcus* sp. CA033	CTCCCCTGCCACTAAGGCGGCGAGTGGGGGCT	20	pMF1	6
SB1671	*Vitiosangium* sp. GDMCC 1.1324	TGCTGCCCGAGCTCGCCTTCGAGGCCCGCGAGCGCC	24	pSa001	6
SE186	*Anaeromyxobacter dehalogenans* 2CP-1	GCTGCCGGGTGGAGCAGGTGCGCTTGCG	16	pSa001	6
SG1033	*Minicystis rosea* strain DSM 24000	TACGCGTGCTCGAGCGGCTCTAGCTGCAGGAGCC	22	pSa001	6
SC3916	*Corallococcus* sp. M34	TGCAGAGCCGGCGCGTGCGCTGAGGCA	15	pSa001	6
SB331	*Myxococcus stipitatus* DSM 14675	ACTACCGCTCGCCTCTCTTACTGCTCGAGCAGCC	30	pYRE	2
SB313	*Myxococcus stipitatus* DSM 14675	CGCCGCTGTCCTCGAGGCCCTTCTCGCTCACGCTAC	28	pYRE	4
SB3625	*Myxococcus* sp. SCHIC003	ATGCCCGTCGTACACCCGGTGCAACAGTCACTCGTG	28	pYRE	4
SC2314	*Myxococcus* sp. AB036A	GTTAGAAGCCTAACGGTCGGCTCTTCCTTCAG	24	pYRE	4
SB317	*Myxococcus stipitatus* DSM 14675	GTTGTCTTCTTGACCAGTTGCATCCTTGGGTTTTGGGTT	29	pYRE	5
SB1721	*Corallococcus* sp. H22C18031201	AGCTACGAGACCACTCGGCGCTCCCACGTCGTCGC	25	pYRE	5
SB3633	*Myxococcus* sp. SCHIC003	ACGCACATCCTCTCCCCCTTCGGAAGCACCAACCTCAACTT	31	pYRE	5
SC2065	*Myxococcus* sp. AB025B	TCCGTGGCCCGCACTGTCGCGACAACCGACCG	22	pYRE	5
SE365	*Sandaracinus* sp. isolate NAT131	GCTCGAGCAGCCGCTTCCGCCGCCGCTGCAGCT	21	pYRE	6

Supplementary Table 4. A comparison of the genomic targets and genome-targeting spacers in myxobacterial representatives and other representative taxa.

Phylum	Genus	Retrieve strains	Strains with unique spacer	Unique spacers	Genome targets	Genome-target spacers	Genomic targets per strain	Genome-target spacers per strain	The average probability of spacer targeting the genome
Myxococcota	Myxococcus	60	40	3975	8870	2029	222	51	0.510440252
Myxococcota	Sorangium	6	5	503	1764	313	353	63	0.622266402
Cyanobacteria	Anabaena	5	4	386	148	77	37	19	0.199481865
Cyanobacteria	Synechococcus	69	15	992	289	152	19	10	0.153225806
Spirochaetota	Leptospira	128	45	1363	335	205	7	5	0.150403522
Crenarchaeota	Sulfolobus	30	24	3482	1687	824	70	34	0.236645606
Pseudomonadota	Escherichia	3839	475	3137	1366	573	3	1	0.182658591
Pseudomonadota	Pseudomonas	1698	242	3929	6279	1578	26	7	0.401628913
Actinomycetota	Bifidobacterium	261	80	3414	3944	1030	49	13	0.301698887
Bacillota	Streptococcus	1226	385	7287	2001	1190	5	3	0.163304515

Supplementary Table 5. PAM Candidates and the suggested preferred PAMs for each CRISPR type. The PAM (3 nucleotides (nt) in the 5`-flanking of the protospacer) direction is shown as 5` → 3` in the table. The high-confident targets, i.e. with less than or equal to 3 nt mismatches (fetched from the IMG/VR and GenBank-Phage databases), are utilized to extract the candidate PAMs. The PAMs with a ratio of more than 10% are regarded as the preferred PAMs (marked in blue). The preferred PAMs that have the same first two bases (labeled in red) are summed as the degenerated perfectly matched targets. Abbreviations: B, C/G/T; W, A/T; V, A/C/G.

**IMG/VR**															
**C-Type**				**B-Type**				**G-Type**				**E-Type**			
0 nt		≤ 3 nt		0 nt		≤ 3 nt		0 nt		≤ 3 nt		0 nt		≤ 3 nt	
TTC	66.67%	TTC	49.29%	ATG	33.33%	ATG	36.84%	TTC	36.36%	TTC	34.43%	AAG	100%	AAC	21.74%
TTT	16.67%	TTT	10.00%	ATC	16.67%	TTG	13.16%	TTG	27.27%	TTG	16.39%			CAA	13.04%
TTG	5.56%	CTC	6.43%	ATT	16.67%	ACG	7.89%	TTA	18.18%	CTC	4.92%			AAG	8.70%
ACC	5.56%	TTG	5.71%	TTG	16.67%	AGG	7.89%	CTC	9.09%	GTC	4.92%			ATC	8.70%
TCC	5.56%	TCC	4.29%	TCG	16.67%	ATA	5.26%	TCC	9.09%	TTA	4.92%			AGC	4.35%
		CGC	2.86%			ATC	5.26%			ATC	3.28%			AGG	4.35%
**PAM**		ACC	2.14%	**PAM**		AGA	2.63%	**PAM**		GAA	3.28%	**PAM**		AGT	4.35%
TTB	88.89%	GTC	2.14%	WTB	83.33%	AGC	2.63%	TTV	81.82%	TCG	3.28%	AAG	100%	CCA	4.35%
		TGG	2.14%			ATT	2.63%			ACC	1.64%			CGA	4.35%
		ATC	1.43%			CAC	2.63%			ACG	1.64%			CGG	4.35%
		CAC	1.43%			CTG	2.63%			ATG	1.64%			GAG	4.35%
		CGG	1.43%			GTA	2.63%			CAG	1.64%			GCG	4.35%
		CTT	1.43%			GTG	2.63%			CAT	1.64%			GGA	4.35%
		ACA	0.71%			TCA	2.63%			CCG	1.64%			TGA	4.35%
		ATA	0.71%			TCG	2.63%			CTT	1.64%			TGC	4.35%
		ATG	0.71%							GAC	1.64%				

	ATT	0.71%				GGC	1.64%		
	CCC	0.71%				GTA	1.64%		
	CCG	0.71%				GTG	1.64%		
	CTG	0.71%				TAC	1.64%		
	GCG	0.71%				TAT	1.64%		
	GGC	0.71%				TCC	1.64%		
	GGG	0.71%				TTT	1.64%		
	TCA	0.71%							
	TCG	0.71%							
	TGC	0.71%							

GenBank-Phage							
C-Type				B-Type			
0 nt		≤ 3 nt		0 nt		≤ 3 nt	
TTC	100%	TTC	45.61%	TTG	33.33%	TTG	21.74%
		TCC	8.77%	ATT	16.67%	ACG	17.39%
		CTC	8.77%	ATC	16.67%	ATG	17.39%
		ACC	3.51%	ATG	16.67%	ACC	4.35%
		ATC	3.51%	TCG	16.67%	AGA	4.35%
		CGC	3.51%			ATC	4.35%
**PAM**		GGC	3.51%	**PAM**		ATT	4.35%
TTC	100%	TGC	3.51%	WTB	83.33%	CAC	4.35%
		TTG	3.51%			CTG	4.35%
		ACA	1.75%			GCG	4.35%
		AGC	1.75%			GCT	4.35%
		CAC	1.75%			GTG	4.35%
		GGG	1.75%			TCG	4.35%
		GTC	1.75%				
		GTG	1.75%				
		GTT	1.75%				
		TAC	1.75%				
		TTT	1.75%				

Supplementary Table 6. The target design for plasmid interference assays. The spacer-matching protospacer sequences are marked in bold and red. The targets are all designed at the 5`untranslated region before the antibiotic gene *apr*^{R}, under the control of the P*aphII* promoter. The PAM regions before the protospacers are marked in bold and black.

**Target**	**Protospacers on the dsDNA**
TIII	5`-CCCTCGCGCGCCCGTGGACGTGGGCCATCACCCACCACGGGGG-3`<br>3`-GGG**AGCGCGCGGGCACCTGCACCCGGTAGTGGGTGGTGCCCCC**-5`
TC	5`-TTCGCTGGAGCTGTTCCGGCTCGCCCGTGTGCCAGCGAA-3`<br>3`-AAG**CGACCTCGACAAGGCCGAGCGGGCACACGGTCGCTT**-5`
TB	5`- ATGGACCCGGACGGACACCGCAAGCGCGTGCGCGTCGGAAACCAT-3`<br>3`- TAC**CTGGGCCTGCCTGTGGCGTTCGCGCACGCGCAGCCTTTGGTA**-5`

Supplementary Table 7. Strains and plasmids used in this study.

**Strains**	**Description**	**Source**
M. xanthus DK1622	Wild-type strain	D. Kaiser (University of Standford)
M. stipitatus strain YRE12	Wild-type strain isolated by laboratory	This study
DK1622-IBF	Up-regulation of MxI-B by replacing promoter in DK1622	This study
DK1622-ICF	Up-regulation of I-C by replacing promoter in DK1622	This study
DK1622-7283F	Up-regulation of III-B by replacing promoter in DK1622	This study
DK1622-ΔCR	Complete knockout of all three CRISPR-Cas systems.	This study
DK1622-ΔIB	I-B knockout in DK1622	This study
DK1622-ΔIC	I-C knockout in DK1622	This study
DK1622-ΔIIIB	III-B knockout in DK1622	This study
E. coli Top10	F^- *mcr*A Δ(*mrr-hsd*RMS-*mcr*BC) Φ80 *lacZΔM15* Δ*lac*X74 *rec*A1 *ara*Δ139 Δ(*ara, -leu*)7697 *gal*U *gal*K *rps*L (Str^R) *end*A1 *nup*G	Laboratory collection
**Plasmids**	**Main Parts and Characteristics**	**Source**
pBJ113	ColE1 ori, kan^R, *galK*	Laboratory collection
pBJ113-ΔIC	ColE1 ori, kan^R, *galK*, homologous arms for I-C knocking out	This study
pBJ113-ΔIB	ColE1 ori, kan^R, *galK*, homologous arms for MxI-B knocking out	This study
pBJ113-ΔIIIB	ColE1 ori, kan^R, *galK*, homologous arms for III-B knocking out	This study
pBJ113-ΔBB	ColE1 ori, kan^R, *galK*, homologous arms for MxI-B and III-B knocking out	This study
pBJ115	ColE1 ori, kan^R	(1)
pBJ115-eGFP-J23100-ddva	ColE1 ori, kan^R, 1.5 Kb homologous arm of *ddvA*, BBa_J23100-BBa_B0034-eGFP-BBa_B0015	(1)
pBJ115-IBF	ColE1 ori, kan^R, BBa_J23100-BBa_B0034-1.5 Kb homologous arm of Cas6 in MxI-B	This study
pBJ115-ICF	ColE1 ori, kan^R, BBa_J23100-BBa_B0034-1.5 Kb homologous arm of Cas3 in I-C	This study
pBJ115-7283F	ColE1 ori, kan^R, BBa_J23100-BBa_B0034-1.5 Kb homologous arm of *MXAN_7283* in III-B	This study
pSWU30	ColE1 ori, tet^R, Mx8*int*	Laboratory collection (2)
pSWU40-T0	ColE1 ori, apr^R, Mx8*int*, MxI-B target removed	This study
pSWU40-TB	ColE1 ori, apr^R, Mx8*int*, MxI-B target equipped	This study
pSWU40-TC	ColE1 ori, apr^R, Mx8*int*, I-C target equipped	This study
pSWU40-TIII	ColE1 ori, apr^R, Mx8*int*, III-B target TIII1 equipped	This study

pTim	PMF1 ori, ColE1 ori, *apr*^{R},	This study
pTim-S0	PMF1 ori, ColE1 ori, *apr*^{R}, pSH058-BBa_B0034-BBa_B0015	This study
pTim-SB	PMF1 ori, ColE1 ori, *apr*^{R}, pSH058-BBa_B0034-SB artificial array (MxI-B repeat+SB spacer+ MxI-B repeat) -BBa_B0015	This study
pTim-SC	PMF1 ori, ColE1 ori, *apr*^{R}, pSH058-BBa_B0034-SC artificial array (I-C repeat+SC spacer+ I-C repeat) -BBa_B0015	This study
pTim-SBR	PMF1 ori, ColE1 ori, aprR, pSH058-BBa_B0034-SBR artificial array (MxI-B repeat+SBR spacer+ MxI-B repeat) -BBa_B0015	This study

Supplementary Table 8. Primers used in this study.

Primers	Primer sequences (5'-3')	Application
IC-3-F	GTGTACTTGCCCAGGTCATG	qPCR for I-C Cas3
IC-3-R	ATTGGCTCACGTTGCAGAAG	
IC-5-F	CGCAAGTTCGAGGACATGTT	qPCR for I-C Cas5
IC-5-R	TCGTAGAACATGAGGCCCAG	
IC-8-F	AATCCTTCGCAGCCCTATGT	qPCR for I-C Cas8
IC-8-R	TGGTGAACGGAGAGCTGAAT	
IC-1-F	CTGAACAAGGAAGGCGAGTG	qPCR for I-C Cas1
IC-1-R	AGAAGGAGACATGGATGCCC	
IC-A-F	CTCGTGGCCTTCAACCCTT	qPCR for I-C CRISPR
IC-A-R	GGTTGAAACGCTGGCACA	
IIIB-0-F	ACGAGGAGGTGATGTCTTGG	qPCR for III-B MXAN_7283
IIIB-0-R	GTCACGGTTCTCAAGCTCAC	
IIIB-2-F	GTCCATGCTCATCCCCGA	qPCR for III-B Cmr2
IIIB-2-R	CTTCCATGGCACGAACTCAC	
IIIB-4-F	GACGATGTCCTTCCTCTGGG	qPCR for III-B Cmr4
IIIB-4-R	CTTGTTGAAGGTACCCGTGG	
IIIB-C6-F	TTGAAAGTCACAATCCGCGC	qPCR for III-B Cas6
IIIB-C6-R	CTTGGACCTGGGCGTATTCA	
IIIB-A-F	CCTGGAGAAGTACCTGTGCT	qPCR for III-B CRISPR
IIIB-A-R	CCGGCGCTTCAAAATCTCTT	
IB-6-F	TCTCCCAGCAATCTCCAGC	qPCR for MxI-B Cas6
IB-6-R	CTCGACCATGCCTATCTGCT	
IB-3-F	CTGTGGCAGTGGAAAGACG	qPCR for MxI-B Cas3
IB-3-R	AGATGTCGAAGTCCACCTCC	
IB-8-F	CCGCTTCATTCACGACTACC	qPCR for MxI-B Cas8
IB-8-R	AAATGCTCTTGAACCGGCTC	
IB-5-F	CGAAGGGGAATGACGAGAAC	qPCR for MxI-B Cas5
IB-5-R	CATTGATGAGGTGCGTGGAC	
IB-41-F	GGATGTGGCGTTTGGTTTCT	qPCR for MxI-B Cas4/1
IB-41-R	CGTTGTTCTTCCACGTCTCC	
IB-A-F	ATGCTAGCCTTGACACCCTT	qPCR for MxI-B CRISPR
IB-A-R	TACGGTGCCAAGTCATGAGT	
gapA-QF	GCCCTGGAAGAGCCTGAACG	qPCR for GapA
gapA-QR	TGGAGACGATGTGGTGCTTGG	
F1.1-F	GAGCGATGCGGTTGTGCTGGTGA	PCR for pYRE F1
F1.1-R	CTACGCGTACCTCAGCAAAAAGGACACG	
F1.2-F	GGGGTGTCAGGTCCCATGCGGA	PCR for pYRE F2.1
F2.1.1-R	GGCATTGCTGTGCACACAGTCGG	

F2.2.2-F<br>F1.2-R	GTCCTCGGACCTGTAGCGGCCG<br>CCCGCAACCCGCAAGAAGTGACC	PCR for pYRE F2.2
F1.3-F<br>F1.3-R	CATGAGCGAGCCCCACAAATAGGTGG<br>TCCAGTGAGGCCTACTTCATGACCGCA	PCR for pYRE F3
F1.4-F<br>F1.4-R	AGAAGAATCCACGTCCGAATCCCAGGT<br>GCTGGCCTTCTCGTCACTCTCGGAG	PCR for pYRE F4
F1.5-F<br>F1.5-R	CGGAACGCCCAAGGGCCTT<br>TCAGGAGCTGCACAAACTCGCCG	PCR for pYRE F5
F1.6-F<br>F2.6.1-R	CCAGGGGGCCGAACGATGTGC<br>GCTGCCTCAATCGCTTCGCACG	PCR for pYRE F6.1
F2.6.2-F<br>F1.6-R	GGCGTCCTTCAAGTTGAGCCGGTG<br>CCGTGGGGGCTGACTCCACTGAA	PCR for pYRE F6.2
F1.7-F<br>F1.7-R	AGACGAGGACGTCATGCTCGGC<br>CTCCACACCCTCCGCAACTGCGAA	PCR for pYRE F7
ICcas3F-F<br><br>ICcas3F-R	GGTACAGTGCTAGCTCTAGAGAAAGAGGAGAAATACTA<br>GATGCATGCGCACGTGCTC<br>CTTCACCTAGATCCTTTTAACCAGCCCCTGGAAGGCC	homologous arm for promoter replacement of I-C
IBcas6F-F<br><br>IBcas6F-R	TACAGTGCTAGCTCTAGAGAAAGAGGAGAAATACTAGA<br>TGGTGTTTGTTGACCTGCTGTT<br>CTTCACCTAGATCCTTTTAATGGTGTGCGGCCACGCA	homologous arm for promoter replacement of MxI-B
7283CH1-R<br>7283CH1-F	CTTCACCTAGATCCTTTTAAAGAGCTCGCGCTCCCGA<br>GGTACAGTGCTAGCTCTAGAGAAAGAGGAGAAATACTA<br>GATGAGACCACGAGGAGGTGA	homologous arm for promoter replacement of III-B
Mx8-intP2-1<br><br>Mx8-intP2-2	CACTGCCCCTCACCCCTTGGCGGTCGTACGCCAACAGGC<br>CGGGGTCCTCAAGTCCGGC<br>CCAAGGGGTGAGGGGCAGTGACGGGCTTGTGCCAGTCA<br>A	Remove MxI-B target in Mx8*int*
IC up-U<br>IC up-D<br><br>IC down-U<br>IC down-D	GCCTGCAGGTCGACTCTAGAAGGGGCGGCAGAAGTCG<br>GGACTCCAGTGCTCTGGGTGTCTCGTTTCCGTCTGGTGA<br>CCG<br>CACCCAGAGCACTGGAGTCC<br>AAACGACGGCCAGTGAATTCGCGTCCCCTCCATGGAGC	I-C knock out
IB up-U<br>IB up-D<br>IB down-U<br><br>IB down-D	GCCTGCAGGTCGACTCTAGATTGACCTGCGGGGCAAGC<br>TAGATTTCCCCACCAGTTCAAAATCACACAT<br>TGAACTGGTGGGGAAATCTACTGAGCTGCGGGTGAAGG<br>G<br>AAACGACGGCCAGTGAATTCGAGGAGCGGAGACAACTC<br>CCAG	MxI-B knock out
IIIB up-U<br><br>IIIB up-D	GCCTGCAGGTCGACTCTAGAGTCGTTCCGCCAAACATTG<br>AGG<br>CTCTTGAAACGGGAATGGTTCTGGAAAT	III-B knock out

IIIB down-U	AACCATTCCCGTTTCAAGAGGCTTCTCCGTCCTTCTTCTTGTCG	
IIIB down-D	AAACGACGGCCAGTGAATTCGCGAGGTCACGAACCCAGG	
Galk-F	CCGAGCTCGAATTCGGGTGACGGGCAATAAGGGCTGCAC	pTim construction
Galk-R	CTCGCCAGTCGATTGGCTGACATATGGTGCACTCTCAGTACAATCTG	
Apm-R	TCAGCCAATCGACTGGCGAG	
Apm-F	CAGGATGAGGATCGTTTCGCATGTCATCAGCGGTGGAGTG	
aphII-P	GCGAAACGATCCTCATCCTG	
Spe-RBS-td	ACTAGTGAAAGAGGAGAAATACTAGATGGTGAGCAA	
Hind-lac	NNNNAAGCTTGGAATTGTTATCCGCTCACAATTCC	
PMF.15-R	TCACCCGAATTCGAGCTCGGT	
pilAIB1-R	TCACGGCGAGCGGGACCACGGAACGCCGGCAGCAGGCACCGGAAGGTCAGCCGGTGCTC	Artificial array preparation
pilAIB1-F-Hind	NNNNAAGCTTGTCCCGCTCGCCGTGATGCCGAAAGGCGTTGAGCACCGGCTGACCTTCC	
pilAIC1-R	GTTCACGAGGAGCGACGCCTCGGACTGCTTCGAGCGGGCCTGGAACTTGATGTTTCAAC	
pilAIC1-F-Hind	NNNNAAGCTTGTCGCTCCTCGTGAACGAGGGGAGCGTGGGTTGAAACATCAAGTTCCAG	
PilA-HDF	CCGGCTTCACCTTCCGC	Homologous arms of *pilA*
PilA-HDR	GGCGTTCTCCCCGGTC	
SBR-F	nnnnAAGCTTGTCCCGCTCGCCGTGATGCCGAAAGGCGTTGAGCACCACGGAACGCCGGCAGCAGGCACCGGAA	Construction of pTim-SBR
SBR-R	nnnnACTAGTgatatcGTGCTCAACGCCTTTCGGCATCACGGCGAGCGGGACCGGCTGACCTTCCGGTGCCTGCTGCCG	

Supplementary Figure 1. The pipeline for the analysis of endogenous CRISPR-Cas systems in myxobacteria with a few complete genomes and lots of incomplete genomes.

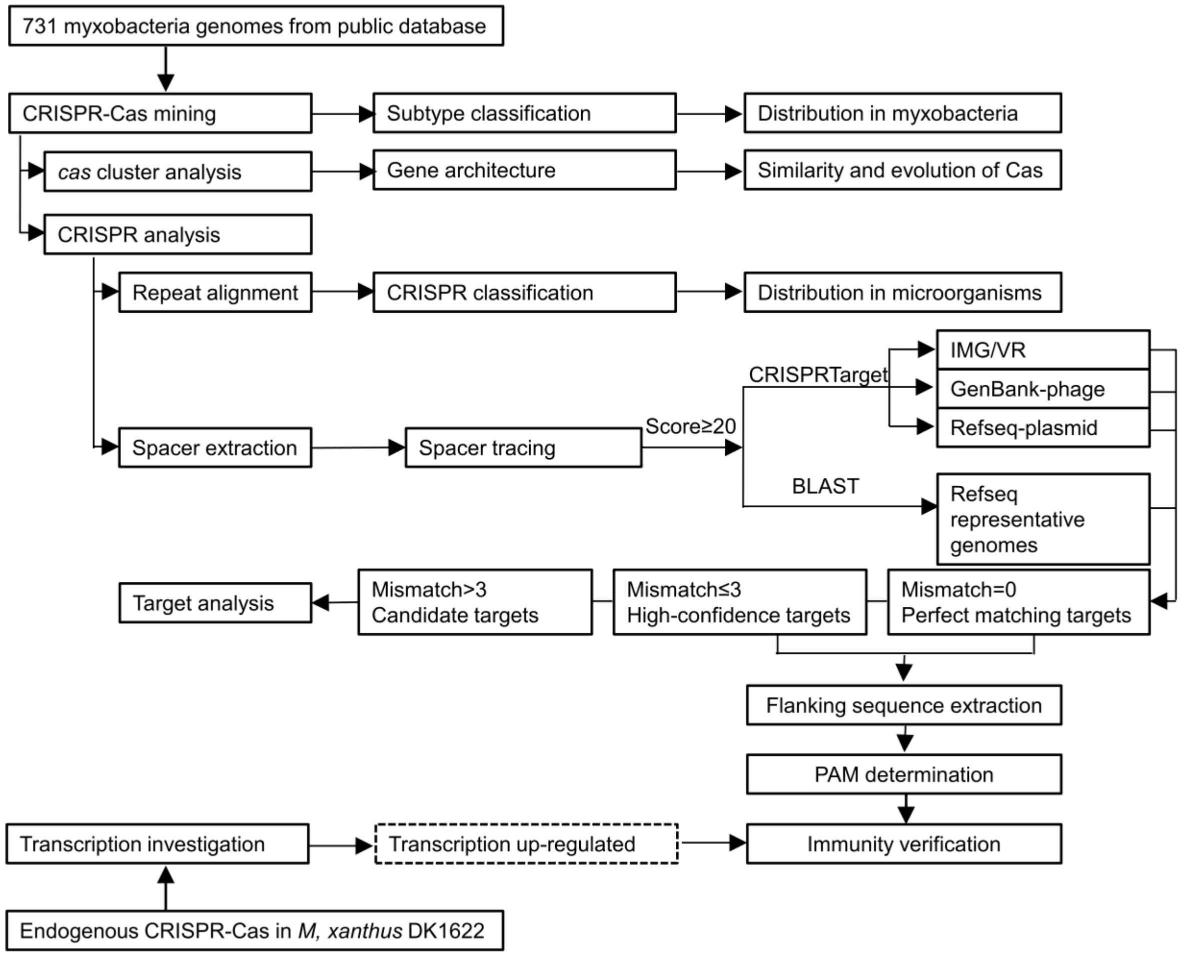

Supplementary Figure 2. The CRISPR-Cas systems retrieved in myxobacterial complete genomes. The genome of *Saccharomyces cerevisiae* S288C (GCF_000146045.2) is used as the outgroup for the phylogenetic tree. Strains without *cas* clusters are marked in bold. Genera containing more than one genome are labeled in the same background color. The two *M. xanthus* DZ2 genomes possess the same number and type of CRISPR-Cas, and the genome GCF_018517205.1 is adopted in the construction of the polygenetic tree while GCF_020827275.1 is not counted in this chart.

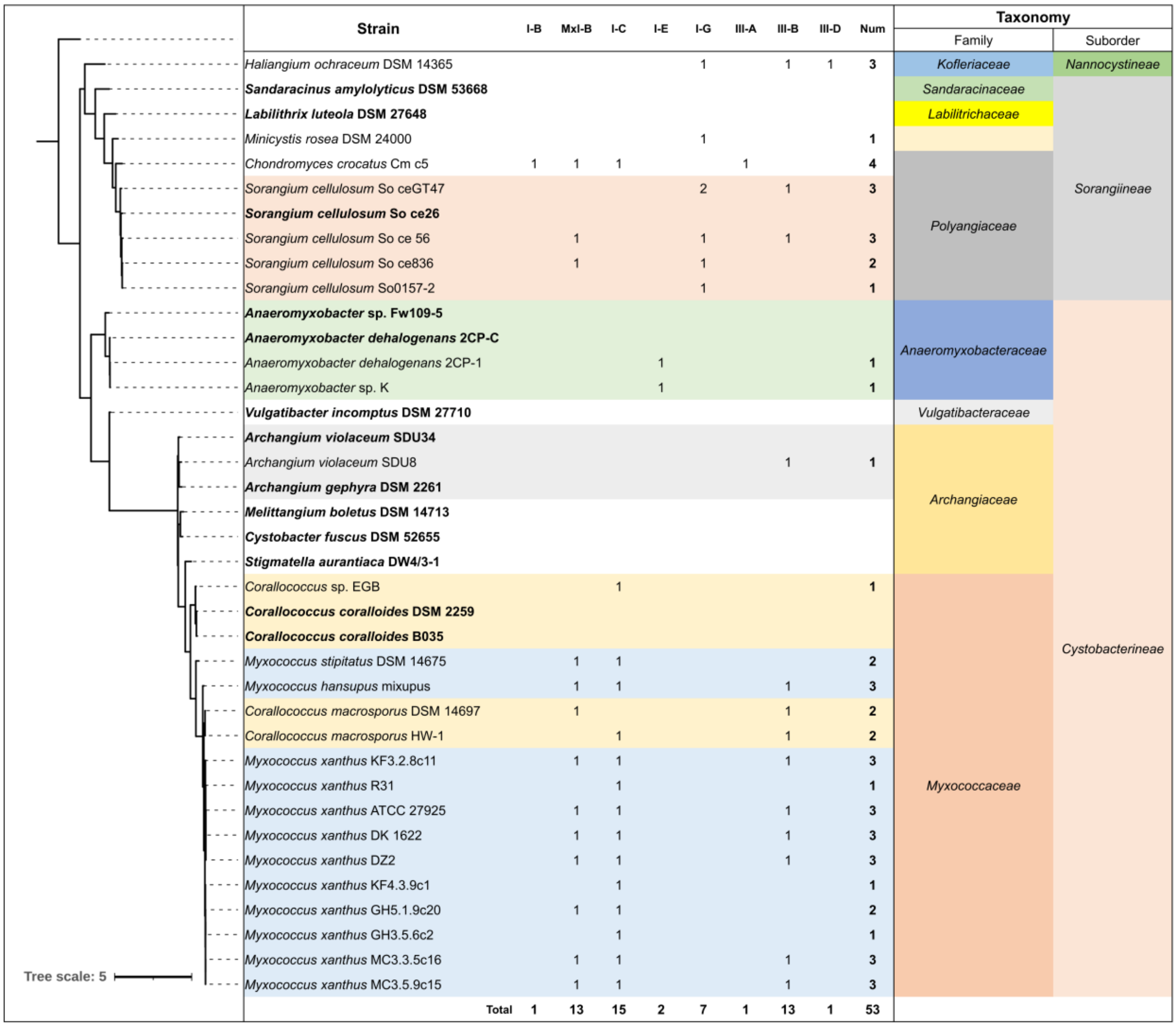

Strain	I-B	MxI-B	I-C	I-E	I-G	III-A	III-B	III-D	Num	Taxonomy: Family	Taxonomy: Suborder
Haliangium ochraceum DSM 14365					1		1	1	3	*Kofleriaceae*	*Nannocystineae*
**_Sandaracinus amylolyticus_ DSM 53668**										*Sandaracinaceae*	*Sorangiineae*
**_Labilithrix luteola_ DSM 27648**										*Labilitrichaceae*	
Minicystis rosea DSM 24000					1				1		
Chondromyces crocatus Cm c5	1	1	1			1			4	*Polyangiaceae*	
Sorangium cellulosum So ceGT47					2		1		3		
**_Sorangium cellulosum_ So ce26**											
Sorangium cellulosum So ce 56		1			1		1		3		
Sorangium cellulosum So ce836		1			1				2		
Sorangium cellulosum So0157-2					1				1		
**_Anaeromyxobacter_ sp. Fw109-5**										*Anaeromyxobacteraceae*	*Cystobacterineae*
**_Anaeromyxobacter dehalogenans_ 2CP-C**											
Anaeromyxobacter dehalogenans 2CP-1				1					1		
Anaeromyxobacter sp. K				1					1		
**_Vulgatibacter incomptus_ DSM 27710**										*Vulgatibacteraceae*	
**_Archangium violaceum_ SDU34**										*Archangiaceae*	
Archangium violaceum SDU8							1		1		
**_Archangium gephyra_ DSM 2261**											
**_Melittangium boletus_ DSM 14713**											
**_Cystobacter fuscus_ DSM 52655**											
**_Stigmatella aurantiaca_ DW4/3-1**											
Corallococcus sp. EGB			1						1	*Myxococcaceae*	
**_Corallococcus coralloides_ DSM 2259**											
**_Corallococcus coralloides_ B035**											
Myxococcus stipitatus DSM 14675		1	1						2		
Myxococcus hansupus mixupus		1	1				1		3		
Corallococcus macrosporus DSM 14697		1					1		2		
Corallococcus macrosporus HW-1			1				1		2		
Myxococcus xanthus KF3.2.8c11		1	1				1		3		
Myxococcus xanthus R31			1						1		
Myxococcus xanthus ATCC 27925		1	1				1		3		
Myxococcus xanthus DK 1622		1	1				1		3		
Myxococcus xanthus DZ2		1	1				1		3		
Myxococcus xanthus KF4.3.9c1			1						1		
Myxococcus xanthus GH5.1.9c20		1	1						2		
Myxococcus xanthus GH3.5.6c2			1						1		
Myxococcus xanthus MC3.3.5c16		1	1				1		3		
Myxococcus xanthus MC3.5.9c15		1	1				1		3		
**Total**	**1**	**13**	**15**	**2**	**7**	**1**	**13**	**1**	**53**		

Supplementary Figure 3. Spacer contents in the CRISPR arrays of different *Myxococcus xanthus* strains having complete genomes. The number on the branches of the left phylogenomic tree indicates the evolutionary distance of the *M. xanthus* strains. Each block in the right represents a spacer, and the gray blocks represent unique spacers. The spacers with the same color mean having the same sequences. The same spacers in different CRISPRs are manually aligned and colored. The long spacer arrays in the same pattern are omitted and displayed using numbers.

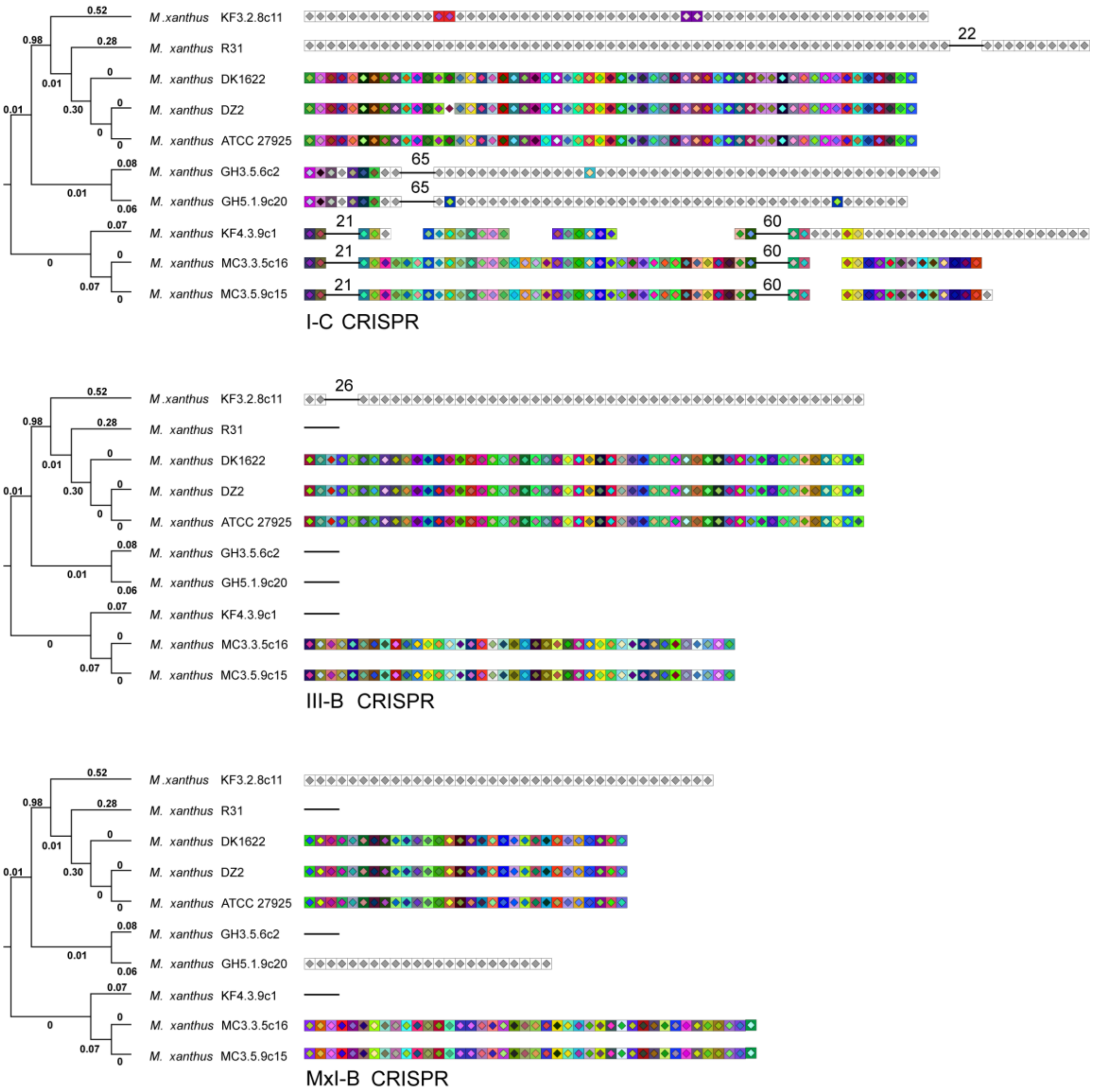

Supplementary Figure 4. Endogenous CRISPR-Cas systems in myxobacteria beyond the seven major subtypes (shown in **Figure 2A**). **(A)** Variants of I-B and III-D in incomplete genomes. Relatively intact CRISPR-Cas clusters are shown in the chart and only one example is listed when several clusters share the same signature proteins and the same gene architectures. The gene size between different clusters is not in proportion. **(B)** Rare subtypes in myxobacteria.

A

B

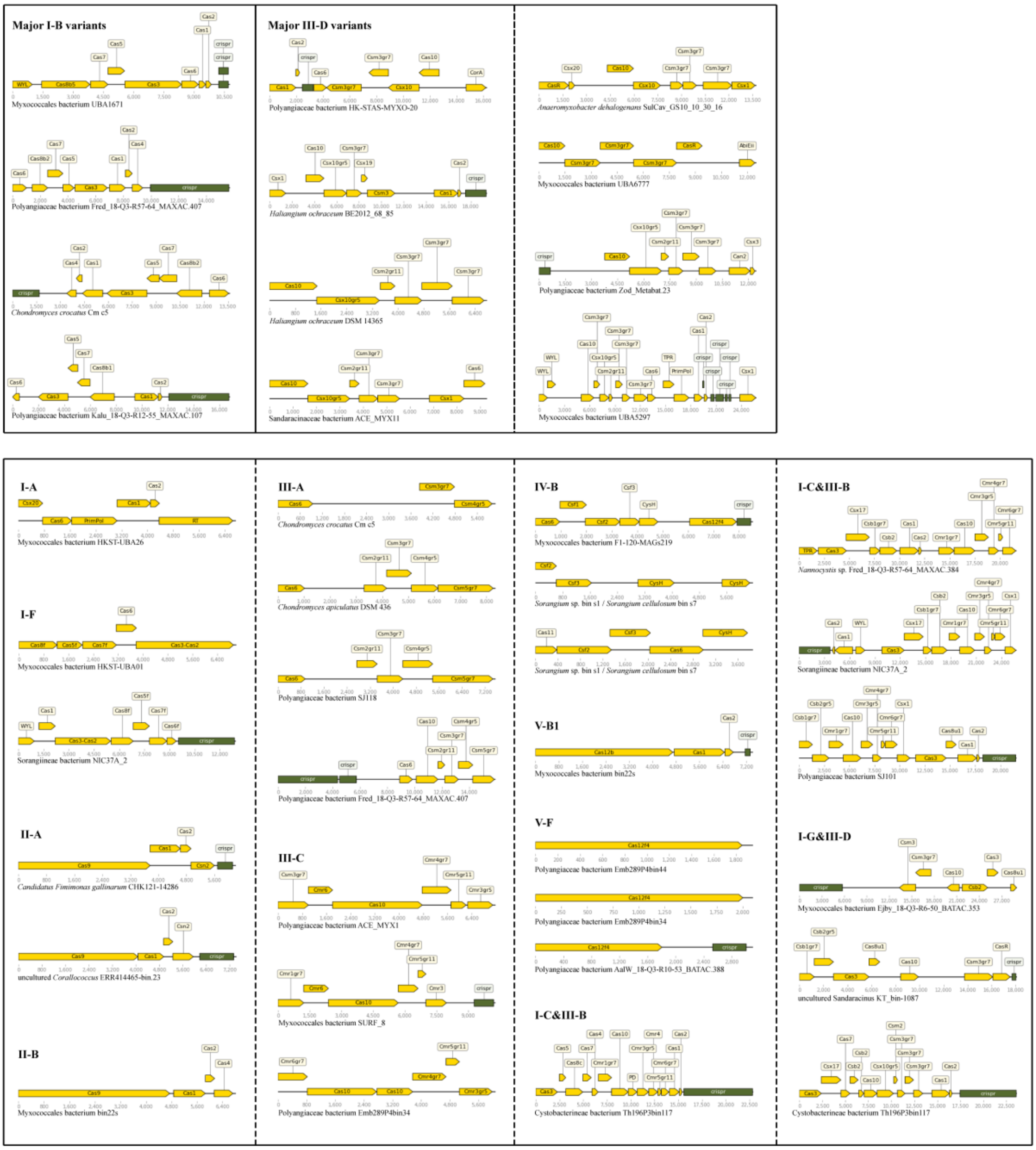

Supplementary Figure 5. Critical residues and motifs of the homologues in every Cas protein.

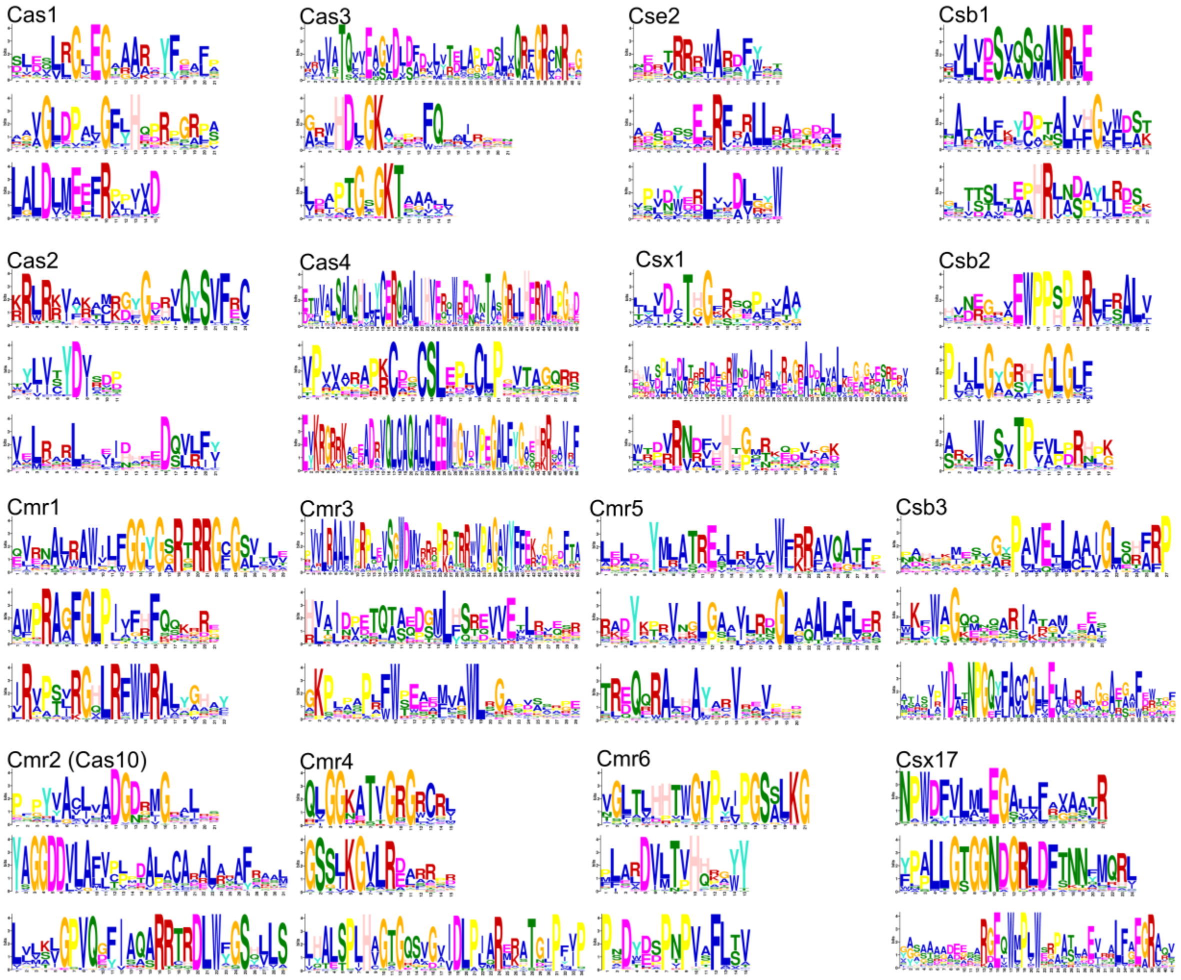

Supplementary Figure 6. B-Type CRISPRs in MxI-B and III-B subtypes. **(A)** The repeat sequences in CRISPRs adjacent to MxI-B and III-B Cas clusters. The different bases are in red. Only the strains containing both MxI-B and III-B are counted. **(B)** The MxI-B and III-B CRISPR-Cas clusters are merged in *M. stipitatus* DSM 14675. Mismatched bases are labeled in red; CR2: CRISPR2; CR3: CRISPR3.

A

Organism name	Consensus Repeat	Repeat Length	Subtype
Corallococcus macrosporus DSM 14697	GTCCCGCTCGCCGTGATGCCGAAAGGCGTTGAGCAC	36	MxI-B
	GTCCCGCTCGCCGTGATGCCGGAAGGCGTTGAGCAC	36	III-B
Myxococcus hansupus mixupus	GTCCCGCTCGCCGTGATGCCGAGAGGCGTTGAGCAC	36	MxI-B
	GTCGCGCTCGCCGTGATGCCGGAAGGCGTTGAGCAC	36	III-B
Myxococcus sp. AB022	GTCCCGCTCGCCGTGATGCCGAAAGGCGTTGAGCAC	36	MxI-B
	GTCCCGCTCGCCGTGATGCCGGAAGGCGTTGAGCAC	36	III-B
Myxococcus sp. AB036A	GTCCCGCTCGCCGTGATGCCGAAAGGCGTTGAGCAC	36	MxI-B
	GTACTGCTCGCCGTGATGCCGGAAGGCGTTGAGCAC	36	III-B
Myxococcus sp. CA005	GTCCCGCTCGCCGTGATGCCGAAAGGCGTTGAGCAC	36	MxI-B
	GTGCCGCTCGCCGTGATGCCGGAAGGCGTTGAGCAC	36	III-B
Myxococcus sp. CA010	GTCCCGCTCGCCGTGATGCCGAAAGGCGTTGAGCAC	36	MxI-B
	GTCCCGCTCGCCGTGATGCCGGAAGGCGTTGAGCAC	36	III-B
Myxococcus sp. CA023	GTCCCGCTCGCCGTGATGCCGAAAGGCGTTGAGCAC	36	MxI-B
	GTCCCGCTCGCCGTGATGCCGGAAGGCGTTGAGCAC	36	III-B
Myxococcus virescens DSM 2260	GTCCCGCTCGCCGTGATGCCGAAAGGCGTTGAGCAC	36	MxI-B
	GTCCCGCTCGCCGTGATGCCGGAAGGCGTTGAGCAC	36	III-B
Myxococcus virescens NBRC 100334	GTCCCGCTCGCCGTGATGCCGAAAGGCGTTGAGCAC	36	MxI-B
	GTCCCGCTCGCCGTGATGCCGGAAGGCGTTGAGCAC	36	III-B
Myxococcus xanthus AB023	GTCCCGCTCGCCGTGATGCCGAAAGGCGTTGAGCAC	36	MxI-B
	GTGCCGCTCGCCGTGATGCCGGAAGGCGTTGAGCAC	36	III-B
Myxococcus xanthus ATCC 27925	GTCCCGCTCGCCGTGATGCCGAAAGGCGTTGAGCAC	36	MxI-B
	GTGCCGCTCGCCGTGATGCCGGAAGGCGTTGAGCAC	36	III-B
Myxococcus xanthus DK 1622	GTCCCGCTCGCCGTGATGCCGAAAGGCGTTGAGCAC	36	MxI-B
	GTGCCGCTCGCCGTGATGCCGGAAGGCGTTGAGCAC	36	III-B
Myxococcus xanthus DSM 16526	GTCCCGCTCGCCGTGATGCCGAAAGGCGTTGAGCAC	36	MxI-B
	GTGCCGCTCGCCGTGATGCCGGAAGGCGTTGAGCAC	36	III-B
Myxococcus xanthus DZ2	GTCCCGCTCGCCGTGATGCCGAAAGGCGTTGAGCAC	36	MxI-B
	GTGCCGCTCGCCGTGATGCCGGAAGGCGTTGAGCAC	36	III-B
Myxococcus xanthus DZF1	GTCCCGCTCGCCGTGATGCCGAAAGGCGTTGAGCAC	36	MxI-B
	GTGCCGCTCGCCGTGATGCCGGAAGGCGTTGAGCAC	36	III-B
Myxococcus xanthus KF3.2.8c11	GTCCCGCTCGCCGTGATGCCGAAAGGCGTTGAGCAC	36	MxI-B
	GTCCCGCTCGCCGTGATGCCGGAAGGCGTTGAGCAC	36	III-B
Myxococcus xanthus MC3.3.5c16	GTCCCGCTCGCCGTGATGCCGAAAGGCGTTGAGCAC	36	MxI-B
	GTCCCGCTCGCCGTGATGCCGGAAGGCGTTGAGCAC	36	III-B
Myxococcus xanthus MC3.5.9c15	GTCCCGCTCGCCGTGATGCCGAAAGGCGTTGAGCAC	36	MxI-B
	GTCCCGCTCGCCGTGATGCCGGAAGGCGTTGAGCAC	36	III-B
uncultured *Corallococcus* ERR414465-bin.23	GTCCCGCTCGCCGTGATGCCGAAAGGCGTTGAGCAC	36	MxI-B
	GTGCCGCTCGCCGTGATGCCGGAAGGCGTTGAGCAC	36	III-B

B

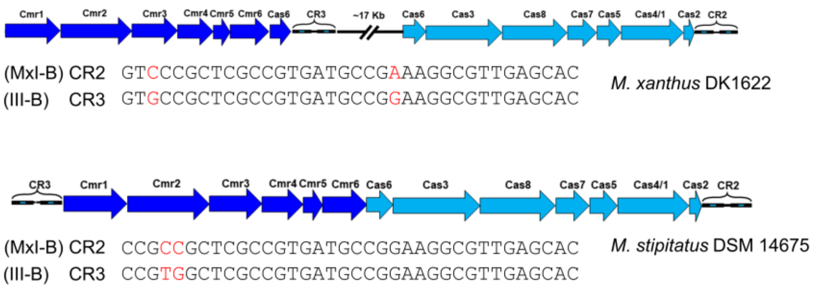

Supplementary Figure 7. Statistics of the traceable spacers in four databases.

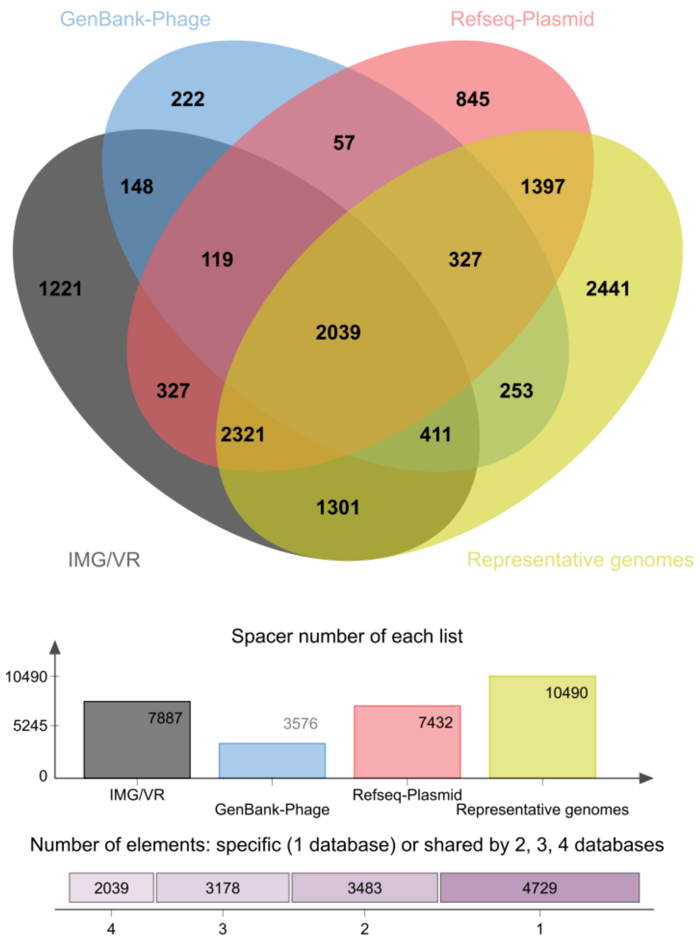

Supplementary Figure 8. Myxobacterial spacers targeting against phages. The gradiently colored blocks mean the number of spacers matching in high confidence (mismatches ≤ 3 nt).

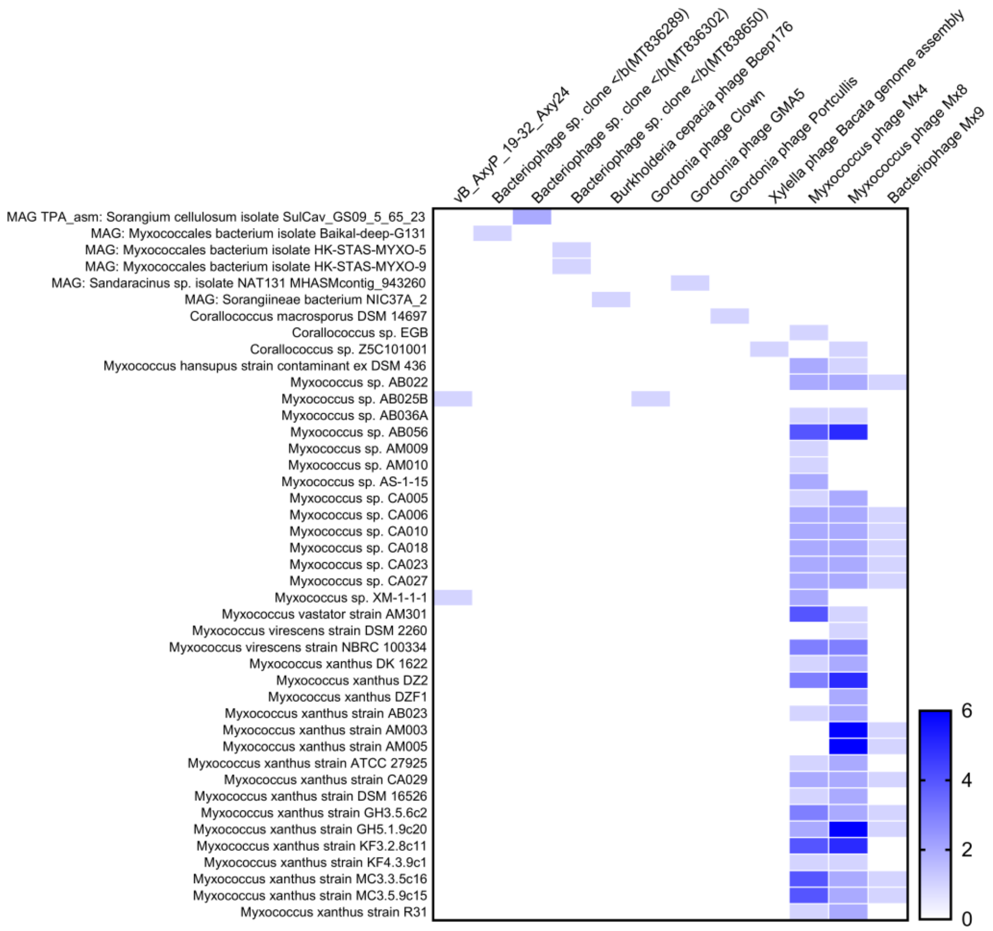

Supplementary Figure 9. PCR verification of the pYRE plasmid. An overlap ~500-bp bases was designed between the neighboring fragments.

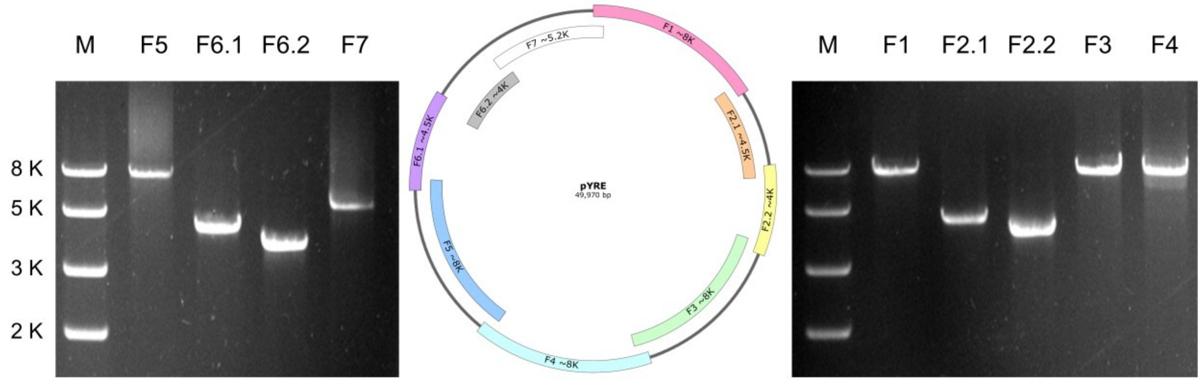

Supplementary Figure 10. PAM determination in non-redundant targets derived from IMG/VR. The 8 nt sequences flanking the protospacers belonging to the same type of CRISPR were extracted and aligned to count PAM sequences.

Mismatch	C-Type		B-Type		G-Type		E-Type	
	5`-flanking	3`-flanking	5`-flanking	3`-flanking	5`-flanking	3`-flanking	5`-flanking	3`-flanking
0 bp	Num=18	Num=18	Num=6	Num=6	Num=11	Num=11	Num=1	Num=1
1 bp	Num=29	Num=29	Num=6	Num=6	Num=10	Num=10	Num=3	Num=3
2 bp	Num=39	Num=39	Num=6	Num=6	Num=8	Num=8	Num=2	Num=2
3 bp	Num=54	Num=54	Num=20	Num=20	Num=32	Num=32	Num=17	Num=17
4 bp	Num=103	Num=103	Num=42	Num=42	Num=63	Num=63	Num=54	Num=54
5 bp	Num=291	Num=291	Num=118	Num=118	Num=143	Num=143	Num=130	Num=130
6 bp	Num=404	Num=404	Num=293	Num=293	Num=524	Num=524	Num=64	Num=64

Supplementary Figure 11. Determination of key motifs for DNA and RNA handling Cas proteins. **(A)** Protein modeling and prediction of key residues for Cas5 and Cas6 using the I-TASSER method. **(B)** Protein modeling and prediction of key residues for Cas3 and Cmr4. **(C)** Alignment of Cas3 and Cas10 protein sequences, showing the MxI-B/I-C Cas3 proteins had the conserved HD motif at the N-terminus, and the III-B Cas10 had the conserved catalytic GGDD motif but lacked the HD motif in *M. xanthus* DK1622. The ligands used in **A** and **B** were obtained from the studied homologous protein models. Cas3: M.xaB, MxI-B in *M. xanthus* DK1622 (ABF91789.1); M.xaC, I-C in *M. xanthus* DK1622 (ABF89301.1); E.co, *E. coli* (WP_000433152.1); P.ae, *P. aeruginosa* (WP_023110258.1); S.mu, *S. mutans* (WP_012997654.1); S.th, *S. thermophilus* (WP_037623090.1); T.te, *Thermobaculum terrenum* (WP_012875832.1); T.th, *Thermus thermophilus* (WP_011229117.1). Cas10: M.xa, *M. xanthus* DK1622 (WP_011557200.1); H.ha, *Halalkalibacterium halodurans* (WP_010896509.1); A.fu, *Archaeoglobus fulgidus* (AIG98864.1); P.fu, *Pyrococcus furiosus* (WP_011012269.1); A.ae, *Aquifex aeolicus* (WP_010880206.1); S.to, *Sulfurisphaera tokodaii* (BAB67074.1); S.so, *Saccharolobus solfataricus* (WP_010923686.1); S.is, *S. islandicus* (WP_016731748.1); P.gi, *Porphyromonas gingivalis* (AAQ66960.1).

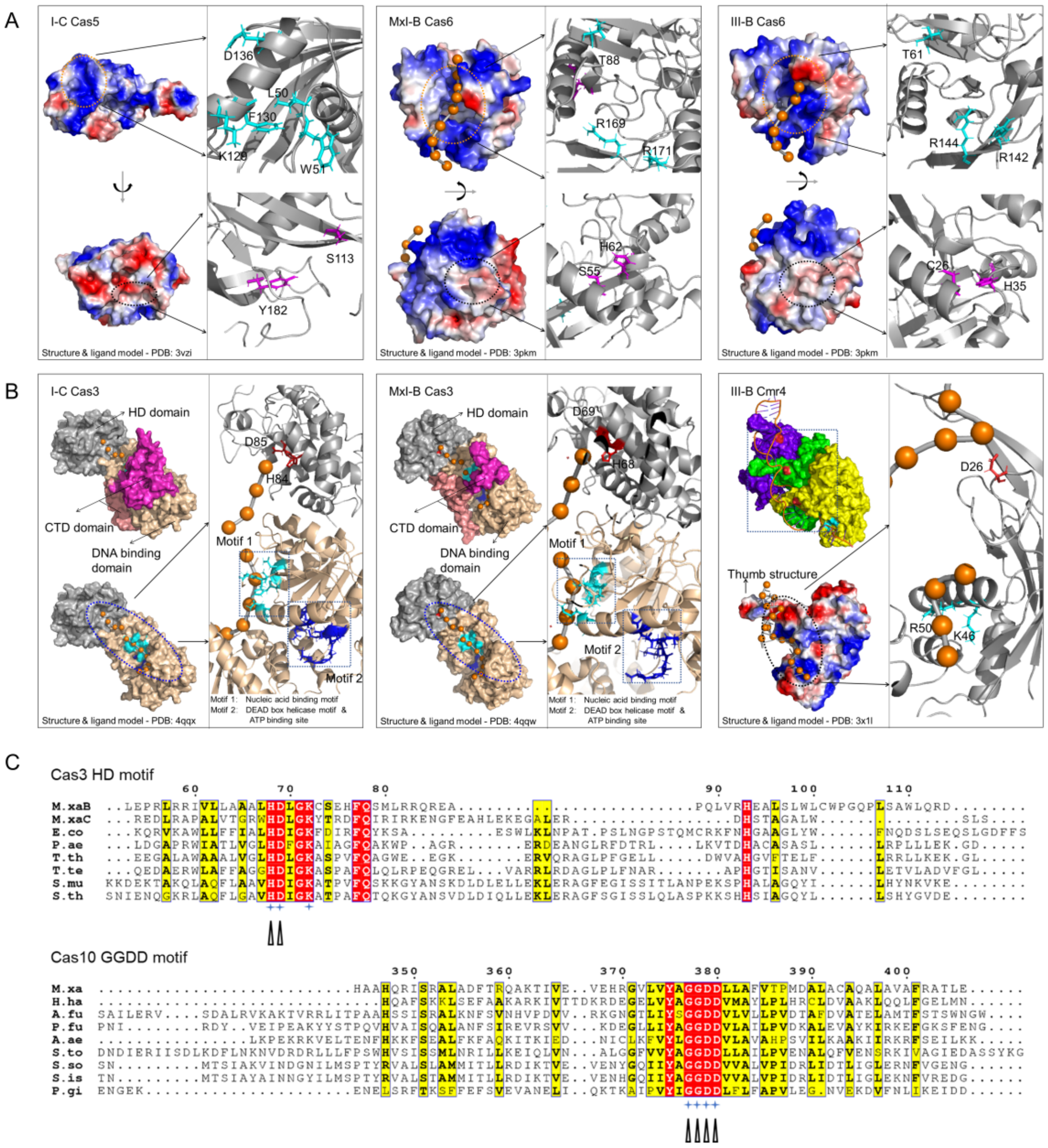
A
I-C Cas5
D136
L50
F130
K129
W51
S113
Y182
Structure & ligand model - PDB: 3vzi
MxI-B Cas6
T88
R169
R171
H62
S55
Structure & ligand model - PDB: 3pkm
III-B Cas6
T61
R144
R142
C26
H35
Structure & ligand model - PDB: 3pkm
B
I-C Cas3
HD domain
CTD domain
DNA binding domain
D85
H84
Motif 1
Motif 2
Motif 1: Nucleic acid binding motif
Motif 2: DEAD box helicase motif & ATP binding site
Structure & ligand model - PDB: 4qqx
MxI-B Cas3
HD domain
CTD domain
DNA binding domain
D69
H68
Motif 1
Motif 2
Motif 1: Nucleic acid binding motif
Motif 2: DEAD box helicase motif & ATP binding site
Structure & ligand model - PDB: 4qqw
III-B Cmr4
D26
Thumb structure
R50
K46
Structure & ligand model - PDB: 3x1l
C
Cas3 HD motif
60
70
80
90
100
110
M.xaB
M.xaC
E.co
P.ae
T.th
T.te
S.mu
S.th
Cas10 GGDD motif
350
360
370
380
390
400
M.xa
H.ha
A.fu
P.fu
A.ae
S.to
S.so
S.is
P.gi

Supplementary Figure 12. Transcription of CRISPR-Cas. **(A)** Distributions of the FPKM values calculated from triplicate RNA-seq data for *M. xanthus* DK1622. **(B)** the semi-quantitative reverse-transcription PCR of the three endogenous CRISPR-Cas systems. Lane 1: MxI-B *cas6*; Lane 2: MxI-B CRISPR array; Lane 3: I-C *cas3*; Lane 4: I-C CRISPR array; Lane 5: III-B *cmr1*; Lane 6: III-B CRISPR array; "GapA+": *gapA* gene (positive control); "GapA-": *gapA* amplification using RNA template (negative control to prove the removal of genome DNA).

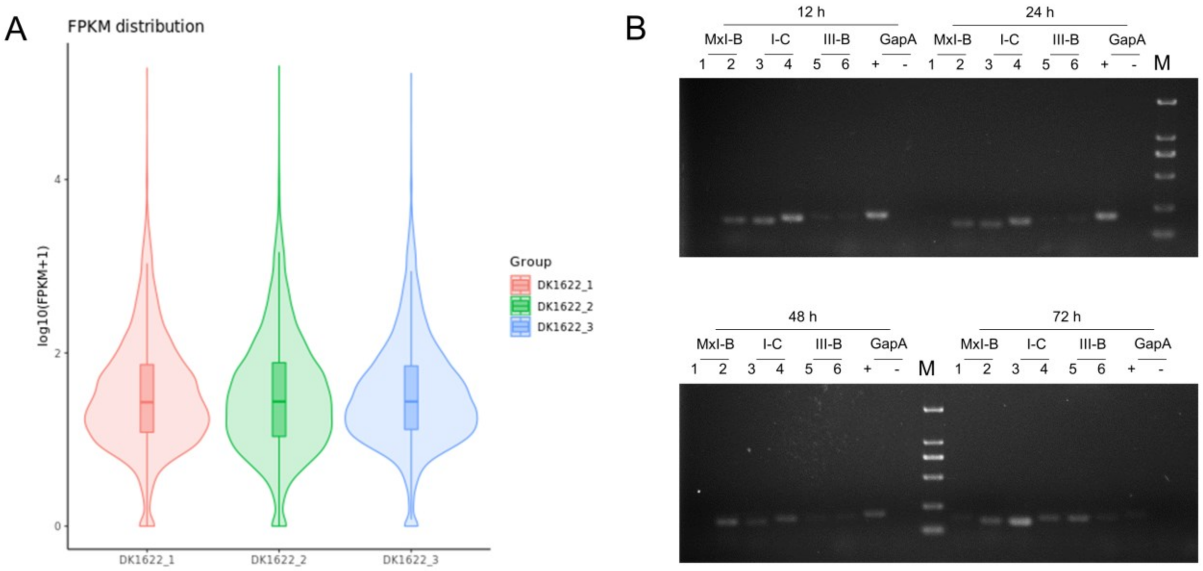

Supplementary Figure 13. Designs for function verification of CRISPR-Cas. **(A)** Diagrams for plasmid interference assays. **(B)** Plasmid design for self-immunity mediated by the promoted CRISPR-Cas systems. If the corresponding system works, the transferred target-containing vectors containing the antibiotic-resistant gene will be degraded, which will decrease the survival of transformants under antibiotic pressure.

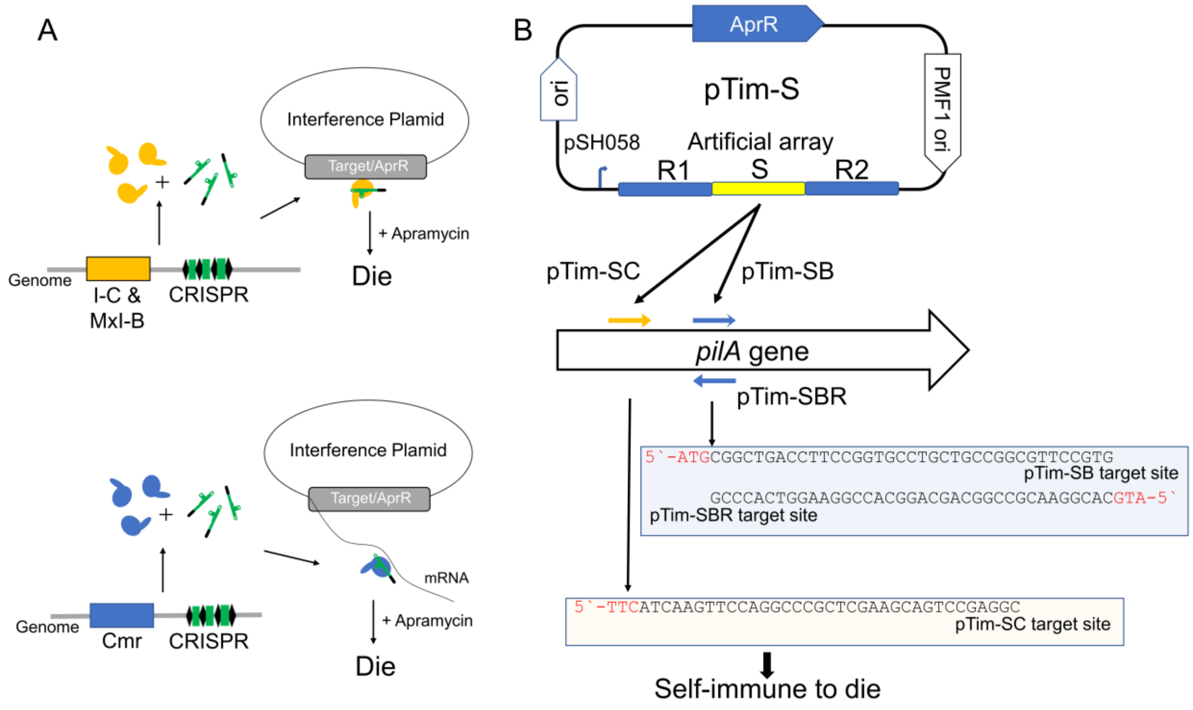

Supplementary Figure 14. Endogenous CRISPR-Cas activation by replacing the native promoters. **(A)** RNA-seq mapping of the three CRISPR-Cas regions before (upper) and after (lower)the up-regulation. The *y*-axis represents the reads count of the transcriptome, and the counts in the continuous region of each CRISPR-Cas gene cluster are significantly increased after replacing the promoter. **(B)** qPCR verification of the activated systems.

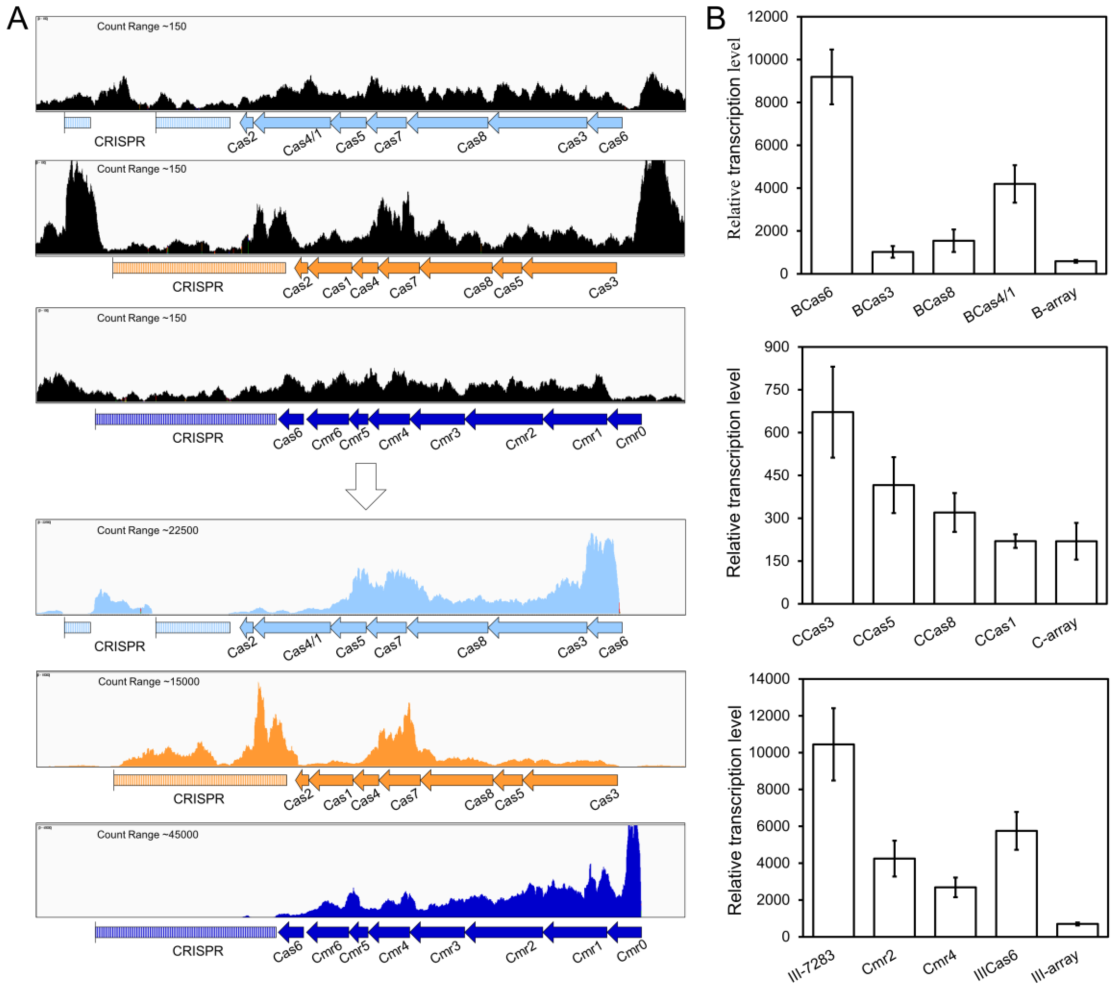

Supplementary Figure 15. Identification of target deletion fragments induced by CRISPR-Cas self-immunity.

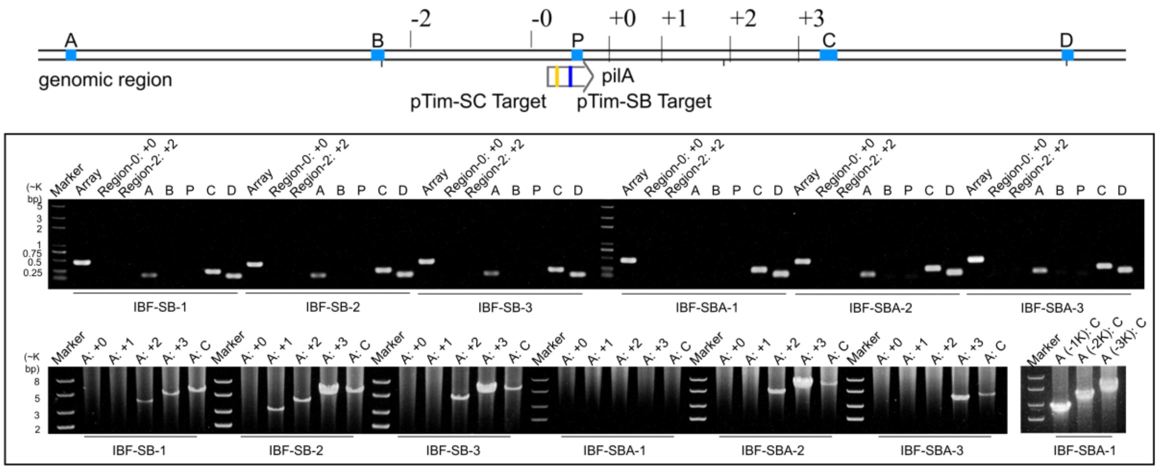

REFERENCES:

1. Hu, W.F., Niu, L., Yue, X.J., Zhu, L.L., Hu, W., Li, Y.Z. and Wu, C. (2021) Characterization of Constitutive Promoters for the Elicitation of Secondary Metabolites in Myxobacteria. *Acs Synth Biol*, **10**, 2904-2909.
2. Wu, S.S. and Kaiser, D. (1997) Regulation of expression of the *pilA* gene in *Myxococcus xanthus*. *J Bacteriol*, **179**, 7748-7758.
